# Engineering Larger Grains in MAPbI_3_ Solar Cells via Covalent Organic Framework (COF)-Assisted Crystallization: High-Performance Devices with Superior Stability Under Prolonged Illumination

**DOI:** 10.3390/polym18202535

**Published:** 2026-10-15

**Authors:** E. M. Mkawi, Saif M. H. Qaid, Abdullah S. Aldwayyan, Prastika Krisma Jiwanti, Chia Ching Kee, Sok Kuan Wong, E. Bekyarova, Yew Hoong Wong

**Affiliations:** 1 Department of Mechanical Engineering, Faculty of Engineering, Universiti Malaya, Kuala Lumpur 50603, Malaysia; mkawimoiz@um.edu.my; 2 Physics and Astronomy Department, College of Science, King Saud University, Riyadh 11451, Saudi Arabia; sqaid@ksu.edu.sa (S.M.H.Q.); dwayyan@ksu.edu.sa (A.S.A.); 3 Nanotechnology Engineering, Faculty of Advanced Technology and Multidiscipline, Universitas Airlangga, Surabaya 60115, Indonesia; prastika.krisma@ftmm.unair.ac.id; 4 School of Engineering, Faculty of Engineering and Technology, Sunway University, Bandar Sunway, Petaling Jaya 47500, Selangor, Malaysia; chiachingk@sunway.edu.my; 5 Department of Pharmacology, Faculty of Medicine, Universiti Kebangsaan Malaysia, Jalan Yaacob Latif, Bandar Tun Razak, Cheras, Kuala Lumpur 56000, Malaysia; 6 Department of Chemistry, University of California at Riverside, Riverside, CA 92521, USA; elena.bekyarova@ucr.edu

**Keywords:** MAPbI_3_, covalent organic frameworks, perovskite solar cells, crystallization kinetics, defect passivation, photoluminescence, long-term stability

## Abstract

Methylammonium lead iodide (MAPbI_3_) is a benchmark absorber for low-cost, high-efficiency perovskite solar cells (PSCs), but its practical deployment is constrained by small grain sizes, high defect densities, and moisture-sensitive surfaces that limit both efficiency and stability. Here, we demonstrate that covalent organic frameworks (COFs) built from commercially available building blocks provide a powerful, multifunctional route to overcome these limitations by simultaneously regulating crystallization, passivating defects, and tuning surface properties in MAPbI_3_ thin films. Three hydrazone-linked and β-ketoenamine frameworks, denoted DMTH-COF, Boc-PDA-COF and Tp-COF, were prepared by condensing 2,5-dimethoxyterephthalohydrazide (DMTH), Boc-PDA-phenylenediamine (Boc-PDA), and 1,3,5-triformylphloroglucinol (Tp), respectively, and were incorporated at low loading into the MAPbI_3_ precursor solution. X-ray diffraction and FE-SEM reveal that all three additives preserve the tetragonal perovskite phase while enlarging the grain size from a few hundred nanometers in pristine films to about 1 μm for DMTH-modified layers, accompanied by narrowed diffraction peaks and reduced microstrain. Raman and FTIR spectroscopy show COF-induced modifications of Pb–I and CH_3_NH_3_^+^ vibrational modes, indicating specific coordination and hydrogen-bonding interactions that stabilize the lattice and reduce trap density. UV-Vis spectra confirm strong visible-light absorption at an unchanged optical bandgap of 1.56 eV across all films, while steady-state PL and EQE measurements evidence enhanced radiative efficiency and improved photon-to-current conversion, particularly at longer wavelengths. X-ray photoelectron spectroscopy confirms that the framework is retained within the film after annealing, the modified absorber showing a N 1s component at 399.7 eV that is absent from the pristine reference, while the Pb 4f and I 3d core levels shift in parallel with their spin–orbit splittings preserved and no metallic lead detected. These material-level improvements translate into substantial device gains in n-i-p PSCs (FTO/c-TiO_2_/m-TiO_2_/COF:MAPbI_3_/Spiro-OMeTAD/Au). Relative to pristine MAPbI_3_ cells (PCE ≈ 14.7%), COF-modified devices exhibit higher Voc, Jsc, and FF, with DMTH-COF-modified cells achieving Voc = 1.183 V, Jsc = 22.98 mA cm^−2^, FF = 76.46%, and PCE = 20.78%, alongside reduced series resistance, increased shunt resistance, and improved device-to-device reproducibility. Water-contact-angle and EDX analyses confirm that COF addition increases surface hydrophobicity and preserves near-ideal Pb:I stoichiometry, supporting enhanced environmental stability. This study introduces bulk COF doping as a simple, scalable strategy to engineer MAPbI_3_ microstructure, energetics, and surface chemistry in a single processing step, and identifies DMTH-COF as the most effective of the three frameworks. More broadly, it positions COF–perovskite hybrids as a tunable platform for high-efficiency, durable perovskite optoelectronics.

## 1. Introduction

Organic–inorganic halide perovskite solar cells (PSCs) have emerged as one of the most promising photovoltaic technologies, driven by their rapid increase in power conversion efficiency (PCE) and compatibility with low-cost solution processing [1,2]. The archetypal perovskite absorber adopts the general formula ABX_3_, where A is an organic cation, B is a divalent metal cation, and X is a halide anion [3]. Among these, methylammonium lead triiodide (MAPbI_3_, MA^+^ = CH_3_NH_3_^+^) has been extensively studied due to its favorable tetragonal phase at room temperature, strong visible-light absorption, long carrier diffusion lengths, and suitable bandgap for single-junction photovoltaic architectures [4]. As a result, single-junction PSCs have achieved certified PCEs of up to 26.1%, positioning them as competitive alternatives to conventional silicon-based solar cells [5].

The outstanding efficiencies of PSCs are rooted in several intrinsic advantages of the perovskite absorber: large absorption coefficients, high charge-carrier mobilities, weakly bound excitons that readily dissociate into free carriers, and long minority carrier diffusion lengths [6]. However, these benefits are severely compromised by structural imperfections that arise during film formation. MAPbI_3_ films typically contain a range of defects including undercoordinated halide ions, undercoordinated Pb^2+^ centers, Pb clusters, Pb–I antisite defects (PbI_3_), and vacancies of I^−^ or MA^+^, which act as non-radiative recombination centers and accelerate degradation [7,8,9]. In polycrystalline films, small grain sizes and high densities of grain boundaries exacerbate recombination losses, impede charge transport, and ultimately limit both PCE and operational stability [10]. Importantly, the density and distribution of these defects are closely linked to the nucleation and crystal-growth dynamics during solution processing [11]. The rapid and often uncontrolled crystallization of MAPbI_3_ from precursor solutions tends to generate heterogeneous nucleation, mosaic domains, and abundant grain boundaries [12]. These microstructural flaws degrade optoelectronic quality and facilitate defect migration and ion transport. Under illumination, the photoinduced strain and thermal expansion of the MAPbI_3_ lattice can further reduce the activation energy for the halide-vacancy migration, thereby further facilitating the ion migration and performance degradation over time [13]. Therefore, the control of the crystallization kinetics and grain growth of perovskite is central to obtaining high-quality films with reduced defect densities, larger grain sizes, and strong operational stability.

To address these challenges, a variety of additive-engineering and interface-modification strategies have been explored. In the precursor solution or at the interfaces, functional small molecules, polymers, ionic liquids and inorganic salts have been incorporated to tune crystallization, passivate defects and stabilize the perovskite phase [14]. A broad range of additives has been investigated to regulate the formation of MAPbI_3_ films and reduce losses associated with structural and electronic defects. These additives can be broadly grouped according to their chemical nature and function. Lewis-basic compounds, including thiourea, urea, and sulfoxide derivatives, interact with Pb^2+^ ions in the precursor solution. Such coordination can alter intermediate-phase formation, slow crystallization, and promote the development of larger and more uniform grains [15]. Ammonium and alkylammonium halides are commonly introduced to compensate for halide-related defects and passivate electrically active sites at grain boundaries and interfaces, often resulting in improved open-circuit voltage [16]. Polymers such as polyethylene glycol (PEG), polyvinylpyrrolidone (PVP), and poly(methyl methacrylate) (PMMA) can influence nucleation and grain growth while improving film coverage and providing partial protection against moisture. Ionic liquids containing imidazolium or pyridinium cations offer another approach because their low volatility and ionic character allow them to modify precursor properties and interact with charged defects in the perovskite layer [17]. Inorganic salts, particularly alkali-metal halides and pseudohalide compounds, have also been employed to regulate crystallization, passivate defects, and limit ion-migration-related losses [18]. Although these additive families operate through different pathways, they share the objective of improving film quality, reducing non-radiative recombination, and stabilizing device operation. Their development has contributed substantially to the progress of MAPbI_3_ solar cells, with reported efficiencies increasing from below 15% to more than 21% during the past decade [19].

Despite substantial advances in additive engineering, several persistent limitations continue to restrict the development of broadly applicable molecular design principles. One important issue is the functional specificity of many reported additives. Compounds selected to regulate nucleation and crystal growth do not necessarily passivate the electronic defects remaining in the deposited film [20]. A second limitation concerns the restricted structural tunability of conventional small-molecule additives. Even minor changes to their functional groups or molecular backbones can substantially alter solubility, volatility, thermal stability, and compatibility with the perovskite precursor solution. Consequently, it is difficult to vary one molecular parameter while keeping the remaining physicochemical properties unchanged [21]. Third, practical drawbacks are also associated with several established additive classes. Although ionic liquids can offer strong chemical interactions and favorable processing characteristics, they are often expensive, and poorly immobilized ionic components may contribute to field-driven redistribution and scan-dependent device behavior. Polymeric additives can improve film coverage and mechanical integrity, but excessive incorporation of electrically insulating polymer chains may obstruct charge transport or accumulate at grain boundaries. Similarly, hygroscopic salts, including some lithium- and cesium-containing compounds, can increase the sensitivity of the perovskite layer to ambient moisture and thereby compromise long-term stability [22]. These limitations do not preclude the use of such materials, but they emphasize the need to optimize additive concentration, chemical stability, spatial distribution, and electronic compatibility rather than considering defect passivation alone. Differences in precursor chemistry, deposition conditions, interfacial layers, and measurement protocols further obscure relationships between molecular structure, film formation, defect passivation, and photovoltaic performance. Comparative investigations conducted under otherwise identical conditions are therefore essential for determining which structural features govern additive–precursor interactions and for establishing reliable design rules for multifunctional additives.

Cost and compatibility with scalable manufacturing must also be considered when evaluating the practical relevance of an additive strategy. In many current perovskite solar-cell architectures, the absorber accounts for only a relatively small proportion of the total materials cost, whereas major contributions arise from the hole-transport layer, transparent conducting substrate, and metallic electrode. Accordingly, additives introduced at the low concentrations typically required for crystallization control or defect passivation are unlikely to increase absorber cost substantially. This advantage, however, depends on the use of readily available compounds with simple preparation and purification requirements. Additives that rely on multistep synthesis, specialized reagents, or laborious isolation may offer excellent laboratory-scale performance while remaining unsuitable for large-area production. An effective additive should therefore combine multiple functions, structural tunability, low loading, solution-process compatibility, and economical availability without introducing new pathways for ionic instability, transport losses, or environmental degradation.

While these strategies have led to significant improvements in efficiencies, they frequently lack structural precision and molecular-level tunability. Covalent organic frameworks (COFs) have emerged as highly attractive candidates for rational control of perovskite crystallization and defect chemistry in this regard [23]. COFs are crystalline, porous, organic polymers constructed via strong covalent bonds between predesigned building blocks, giving rise to periodic architectures with well-defined pores and extended π-conjugation [24]. Their low density, large surface area, tunable pore environments, and chemically programmable frameworks make COFs naturally attractive for applications such as photocatalysis, energy storage, and photovoltaics [25]. In perovskite systems in particular, they can play several complementary roles: they provide structural scaffolds that template nucleation and guide crystal growth, they act as electronic modulators that assist charge separation and transport, and they form physical barriers that help to suppress ion migration and protect the material from environmental degradation [26]. For instance, in situ formation of 2D COFs within PbI_2_ layers has been shown to regulate perovskite crystallization, lower defect densities, and enhance carrier transport. Other studies have demonstrated that carefully chosen COF–perovskite composites can suppress phase degradation (e.g., δ-phase formation in FAPbI_3_), reduce surface trap states, and significantly improve long-term device stability [27].

Reports on COF–perovskite integration may be categorized according to the location and function of the framework in the device. A frequently studied design is a pre-synthesized COF sandwiched as an interlayer between the perovskite absorber and a charge-transport layer. Lewis-basic groups on the pore surface may interact with undercoordinated Pb^2+^ sites, minimizing interfacial defects and the accompanying non-radiative recombination in this configuration [28]. For example, Mohamed MG et al. sandwiched two-dimensional Car-ETTA and TFPPy-ETTA COFs between PTAA and the perovskite layer in inverted solar cells [29]. Their porous surfaces promoted perovskite nucleation and crystal growth, resulting in better morphology and crystallinity of the films. The Car-ETTA- and TFPPy-ETTA-based devices gave PCEs of 19.79% and 19.72%, respectively, while the control gave 17.40%. Guo J et al. later utilized sulfhydryl-functionalized COFs (HS-COFs) at both of the perovskite film interfaces [30]. The COF at the buried interface was beneficial in reducing the residual lattice strain and enhancing the resistance to UV exposure, whereas at the top interface, the sulfhydryl groups passivated the surface defects via S–Pb interactions. This dual interface method achieved a PCE of 24.26%, which is one of the highest values recorded for COF-modified perovskite solar cells.

The second technique is to directly introduce COFs into the perovskite absorber as bulk additives. Wu et al. reported a conjugated three-dimensional framework, SP-3D COF, to realize good electrical coupling with the perovskite [31]. The suggested bidirectional charge-transfer process improved carrier transport and enhanced the PCE from 16.70% to 19.07%. Nie et al. improved both film formation and operational stability by using TTDA-TTA COF synthesized from thieno [3,2-b]thiophene-2,5-dicarbaldehyde and 2,4,6-tris(4-aminophenyl)-1,3,5-triazine [32]. The expanded conjugated structure and π–π interactions boosted perovskite crystallization and suppressed degradation in humid and hot settings, achieving a champion PCE of 23.35%. In a similar fashion, Gao X et al. used an amino-functionalized framework (NH_2_-COF) to control crystallization and passivate defects [15]. The coordination of amino groups and Pb sites enhanced the crystallinity of the film and decreased non-radiative recombination, leading to a champion PCE of 22.13%. These investigations suggest that COFs may enhance perovskite devices by tailoring key interfaces or by regulating crystallization, defect chemistry and charge transport in the absorber.

Two limitations of this body of work motivate the present study. First, the framework is in most cases deposited as a discrete layer or applied as a post-treatment, which retains the process-complexity penalty discussed above and confines the interaction to the surface of the absorber rather than to the grain boundaries distributed throughout it. Reports in which COF chemistry is introduced directly into the MAPbI_3_ precursor ink, so that it acts during nucleation and growth rather than after it, remain comparatively few. Despite encouraging results, the existing literature provides limited guidance for the rational design of framework modifiers. Individual studies generally focus on only one framework, making it difficult to determine whether the reported improvements arise from linker coordination, pore architecture, π-conjugation, or simply the incorporation of an organic component at grain boundaries [33]. Comparisons among separate reports do not resolve this issue because perovskite-film properties are highly sensitive to experimental variables, including precursor stoichiometry, antisolvent treatment, and annealing conditions, which vary considerably among laboratories [34]. Moreover, many previous investigations have examined compositions other than MAPbI_3_. These include formamidinium-based absorbers, for which stabilization against the photoinactive δ-phase is a primary concern, and lead-free perovskites studied mainly for photocatalytic applications [35,36]. Because these materials exhibit defect populations and degradation pathways that differ from those of MAPbI_3_, their reported trends cannot be applied directly to MAPbI_3_ devices. In particular, there is a lack of comparative studies that investigate how different COF linkers and functional groups regulate MAPbI_3_ nucleation, grain coalescence, and optoelectronic properties within a unified device platform. Second, almost all published studies examine a single COF system, so the reported improvements cannot be attributed to any particular structural feature of the framework. Without a controlled comparison of chemically distinct frameworks within one device platform, it is not possible to establish which linkage motifs, functional groups, or coordination environments are responsible for the observed effects, and it is therefore not possible to formulate design rules for selecting or synthesizing new candidates.

**Figure 1:**
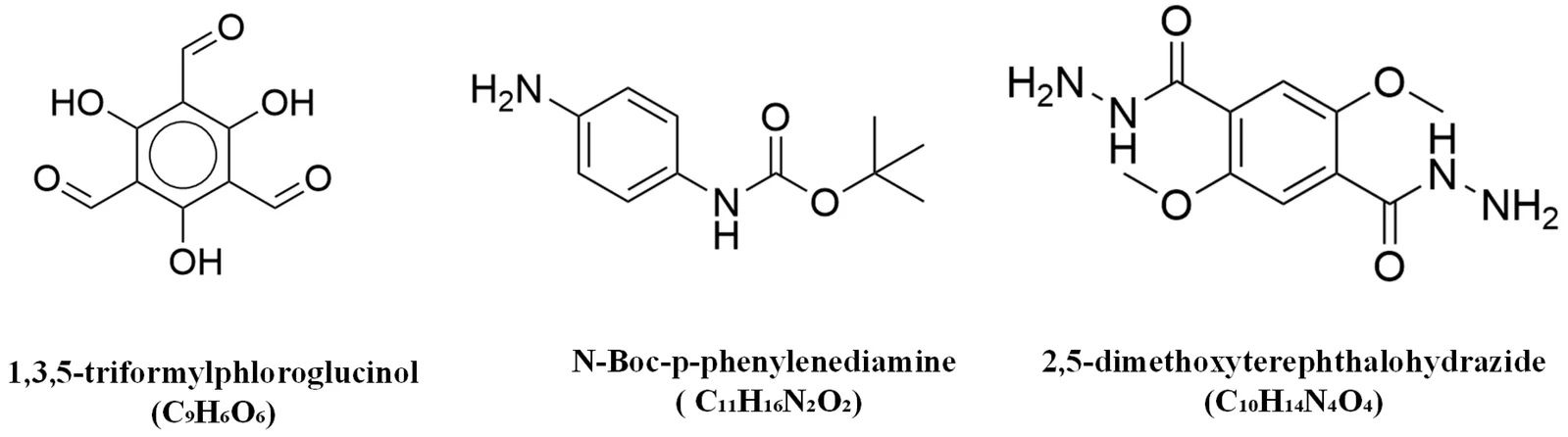
Molecular structures of the building blocks used to prepare the three covalent organic frameworks employed in this work, drawn as two-dimensional skeletal formulas with their molecular formulas: 1,3,5-triformylphloroglucinol (Tp, C_9_H_6_O_6_), Boc-PDA phenylenediamine (Boc-PDA, C_11_H_16_N_2_O_2_) and 2,5-dimethoxyterephthalohydrazide (DMTH, C_10_H_14_N_4_O_4_). Each framework is formed by the solvothermal condensation of Tp with an amine or hydrazide component: Boc-PDA, DMTH, or p-phenylenediamine. Each framework is formed by the solvothermal condensation of Tp with one amine or hydrazide component: Boc-PDA, p-phenylenediamine, or DMTH, giving Boc-PDA-COF, Tp-COF and DMTH-COF, respectively.

The choice of building blocks determines the coordination environment and hydrogen-bonding capacity that the resulting framework can present to the perovskite, and the three used here were selected to vary these systematically while retaining a common structural motif. 1,3,5-Triformylphloroglucinol serves as the trialdehyde node in all three frameworks. Its C_3_-symmetric geometry directs the formation of hexagonally ordered networks, and its three hydroxyl groups, positioned ortho to the aldehyde functions, permit the keto-enol tautomerism that stabilizes β-ketoenamine linkages against hydrolysis, giving frameworks of unusual chemical robustness [37]. Condensation with p-phenylenediamine yields the simplest of the three networks, in which a rigid, minimally functionalized aryl linker provides extended π-conjugation and a high density of nodes but few sites capable of specific interaction with the perovskite precursors. Boc-PDA introduces a bulky carbamate substituent on the same aryl backbone, adding steric volume and carbonyl oxygen atoms that can act as Lewis-basic sites while retaining the aromatic character of the linker [38]. 2,5-Dimethoxyterephthalohydrazide differs more substantially: its two hydrazide groups each provide a carbonyl oxygen and two nitrogen atoms capable of coordination and hydrogen bonding, and the two methoxy substituents are electron-donating and supply further oxygen donors [39]. It therefore presents the richest combination of coordination and hydrogen-bonding motifs of the three, and the highest density of sites available to interact with Pb^2+^ centers and with the methylammonium and iodide species in the precursor solution. Comparing these three frameworks within one device platform thus separates the contribution of the linker chemistry from that of the common node, which is the basis of the structure–property correlation presented in this work. The molecular structures of the three building blocks are shown in Figure 1.

In this work, we address these gaps by systematically engineering MAPbI_3_ crystallization through the incorporation of three distinct COFs, each formed by condensation of 1,3,5-triformylphloroglucinol (Tp) with 2,5-dimethoxyterephthalohydrazide (DMTH), Boc-PDA phenylenediamine (Boc-PDA), and 1,3,5-triformylphloroglucinol (Tp). These building blocks give frameworks with different linkage motifs and pore chemistries, spanning hydrazone and β-ketoenamine linkages, and therefore different electronic environments and coordination sites for the perovskite precursor species. DMTH-COF introduces methoxy-substituted hydrazide units that act as electron-donating and coordinating groups, potentially slowing crystal growth and favoring larger grain formation. Boc-PDA-COF offers protected amine functionalities that can be deprotected to expose reactive sites, allowing controlled interaction with the perovskite lattice. Tp-based frameworks typically form highly ordered, hexagonally porous β-ketoenamine COFs, which can serve as rigid templates for perovskite nucleation. By integrating these COFs directly into the MAPbI_3_ precursor and employing a controlled spin-coating process, we perform a comparative study of how COF chemistry influences crystallization kinetics, grain size, film morphology, defect passivation, optical properties, and device stability. We show that COF-assisted crystallization leads to enlarged grains, reduced grain-boundary density, and suppressed non-radiative recombination, with DMTH-based COFs in particular yielding the most pronounced improvements in film quality and device performance. The resulting mesoscopic n-i-p PSCs exhibit enhanced PCE and markedly improved stability under prolonged illumination and ambient humidity. This work provides strong evidence that rationally designed COFs can serve as powerful crystallization regulators and defect-passivating scaffolds for MAPbI_3_, providing a structurally tunable platform to simultaneously engineer morphology, electronic structure, and stability in perovskite solar cells. The novelty of this study lies in three key aspects. First, we employ COFs as bulk additives within the MAPbI_3_ precursor solution rather than as separately deposited interfacial layers. As set out in the preceding discussion, this distinction is consequential: a bulk additive acts during nucleation and growth and is therefore distributed among the grain boundaries throughout the film, whereas a deposited layer acts only at the surface and requires an additional processing step. Second, we present a systematic comparison of three chemically and structurally distinct hydrazone- and β-ketoenamine-linked COFs, prepared from DMTH, Boc-PDA and p-phenylenediamine with 1,3,5-triformylphloroglucinol, within the same MAPbI_3_ platform, directly correlating framework structure with grain growth statistics, vibrational signatures, optical response, wetting behavior and device-level stability. Third, we show that an optimized COF (DMTH) can simultaneously deliver micrometer-scale grains, reduced microstrain, enhanced hydrophobicity and that the resulting energy-level alignment at the perovskite/HTL interface is consistent with more efficient hole extraction. Together, these advances position COF-based bulk doping as a previously underexplored but highly effective strategy for integrated microstructural and interfacial engineering in perovskite photovoltaics.

## 2. Methodology

### 2.1. Chemicals and Materials

All chemicals were used as received without further purification unless otherwise noted. N-Boc-PDA-phenylenediamine (Boc-PDA, 97%), 2,5-dimethoxyterephthalohydrazide (DMTH, 98%), 1,3,5-triformylphloroglucinol (Tp,95%), lead(II) iodide (PbI_2_, 99.999%), methylammonium iodide (MAI, 99%), dimethyl sulfoxide (DMSO, anhydrous, 99.9%), N,N-dimethylformamide (DMF, anhydrous, 99.8%), mesitylene (98%), acetic acid (glacial, 99.7%), chlorobenzene (99.9%), ethyl acetate (99.8%), 1,4-dioxane (99%), and p-toluenesulfonic acid (PTSA, 98%) were purchased from Sigma-Aldrich (St. Louis, MO, USA). Spiro-OMeTAD (99%), 4-tert-butylpyridine (TBP, 96%), bis(trifluoromethanesulfonyl)imide lithium salt (LiTFSI, 99.95%), acetonitrile (anhydrous, 99.8%), titanium isopropoxide (97%), titanium tetrachloride (99.9%), ethanol (99.8%), acetone (99.5%), isopropanol (99.5%), hydrochloric acid (37%) and zinc powder were obtained from Merck. Mesoporous titania paste (18NR-T) was obtained from Dyesol (Queanbeyan, NSW, Australia), and gold pellets (99.99%) for thermal evaporation from Spiro-OMeTAD (99%), 4-tert-butylpyridine (TBP, 96%), bis(trifluoromethanesulfonyl)imide lithium salt (LiTFSI, 99.95%), acetonitrile (anhydrous, 99.8%), titanium isopropoxide (97%), titanium tetrachloride (99.9%), ethanol (99.8%), acetone (99.5%), isopropanol (99.5%), hydrochloric acid (37%) and zinc powder were obtained from Merck (Merck KGaA, Darmstadt, Germany). Mesoporous titania paste (18NR-T) was obtained from Dyesol, and gold pellets (99.99%) for thermal evaporation from Merck (Merck KGaA, Darmstadt, Germany).

### 2.2. Preparation of TiO_2_ Layers

**Figure 2:**
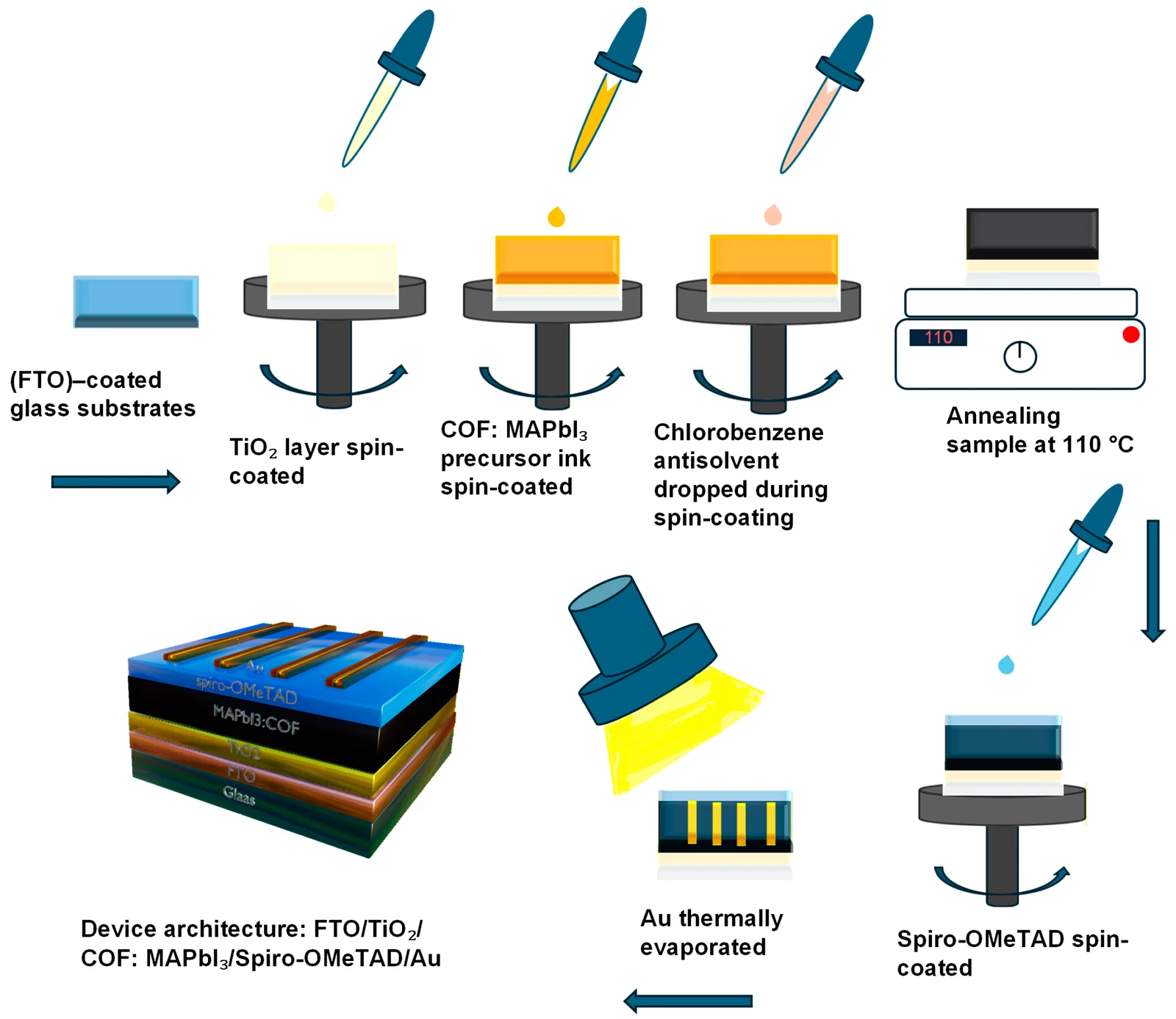
Schematic illustration of the fabrication process for COF-modified MAPbI_3_ perovskite solar cells. A compact and mesoporous TiO_2_ layers were deposited on FTO-coated glass, followed by spin-coating of the COF:MAPbI_3_ precursor ink. Chlorobenzene was applied as an antisolvent during spin-coating, and the films were subsequently annealed at 110 °C. Spiro-OMeTAD was then spin-coated as the hole-transport layer, followed by thermal evaporation of the Au electrode. The final device architecture was FTO/TiO_2_/COF:MAPbI_3_/Spiro-OMeTAD/Au.

Fluorine-doped tin oxide (FTO)–coated glass substrates (sheet resistance 7–10 Ω sq^−1^, Sigma-Aldrich (St. Louis, MO, USA)) were patterned by wet chemical etching using HCl and Zn powder. The substrates were then sequentially cleaned in DI water, acetone, and isopropanol (10 min each, ultrasonic bath) and dried in a vacuum oven at 110 °C for 15 min. A compact TiO_2_ (c-TiO_2_) precursor solution was prepared by adding titanium isopropoxide (380 μL) dropwise into ethanol (2.56 mL) under continuous stirring. In a separate vial, diluted HCl (340 μL, HCl: water = 3:1 v/v) was mixed with ethanol (2.52 mL). The acidic ethanol solution was then added dropwise to the titanium isopropoxide solution under vigorous stirring, followed by an additional 30 min of stirring to obtain a homogeneous, transparent sol. The TiO_2_ precursor was spin-coated onto the cleaned FTO substrates at 2000 rpm for 30 s, as shown in Figure 2. The resulting films were dried at 100 °C and then sintered at 450 °C for 40 min to form the c-TiO_2_ layer. To improve interfacial contact between the compact and mesoporous layers, the c-TiO_2_-coated substrates were immersed in a 40 mM aqueous TiCl_4_ solution at 70 °C for 30 min, rinsed, and annealed at 450 °C for 30 min. The mesoporous TiO_2_ (m-TiO_2_) scaffold was deposited by spin-coating a diluted TiO_2_ paste (Dyesol (Queanbeyan, NSW, Australia) 18NR-T) at 4000 rpm. The paste was first dispersed in ethanol (1 g in 5 mL) by repeated sonication and stirring until a uniform suspension was obtained. The m-TiO_2_-coated substrates were subsequently calcined at 500 °C for 1 h. The patterned FTO substrates gave a device area of 0.50 cm^2^, defined by the overlap of the FTO and Au electrodes; J–V characterization was performed through a metal aperture mask, as described in Section 2.6.

### 2.3. Synthesis of Covalent Organic Frameworks (COFs)

Three COFs were synthesized via solvothermal condensation. These COFs were specifically selected to provide distinct pore chemistries and coordination environments, enabling a systematic investigation of how COF structure directs MAPbI_3_ nucleation and grain growth.

**DMTH-based COF (DMTH-COF).** DMTH (26 mg, 0.102 mmol, 1.43 equiv) and Tp (15 mg, 0.071 mmol, 1.00 equiv) were dissolved in a mixture of mesitylene (4 mL), 1,4-dioxane (2 mL) and acetic acid (0.6 mL) in a glovebox. The mixture was sonicated for 10 min, transferred to a sealed vessel and heated at 140 °C for 72 h (28.3 mg, 77%). The resulting yellow precipitate was collected by centrifugation, washed with ethanol (4 × 10 mL) and acetone (4 × 10 mL), and dried under vacuum at 75 °C for 12 h.

**Boc-PDA-based COF (Boc-PDA-COF).** Boc-PDA (30 mg, 0.144 mmol, 1.38 equiv), Tp (22 mg, 0.105 mmol, 1.00 equiv) and PTSA (2 mg, 0.012 mmol, 0.11 equiv) were combined in 1,4-dioxane (2 mL) and mesitylene (4 mL) in a glovebox. After sonication and degassing, the mixture was heated at 100 °C for 48 h (26.7 mg, 87%). The precipitate was isolated by centrifugation, washed with ethanol (4 × 10 mL) and acetone (4 × 10 mL), and dried under vacuum at 75 °C for 12 h.

**Tp-based COF (Tp-COF).** p-Phenylenediamine (16.2 mg, 0.150 mmol, 1.43 equiv) and Tp (22 mg, 0.105 mmol, 1.00 equiv) were dissolved in mesitylene (4 mL), 1,4-dioxane (2 mL) and acetic acid (0.3 mL) in a glovebox. After 10 min of sonication and degassing the mixture was heated at 120 °C for 72 h. The precipitate was collected by centrifugation, washed with ethanol (4 × 10 mL) and acetone (4 × 10 mL), and dried under vacuum at 75 °C for 12 h (25.4 mg, 80%).

The three frameworks were prepared by condensation with 1,3,5-triformylphloroglucinol (Tp). Those formed with DMTH and Boc-PDA are denoted DMTH-COF and Boc-PDA-COF after the hydrazide or amine partner, while the framework formed with p-phenylenediamine is denoted Tp-COF. For brevity, perovskite films and devices containing them are labeled MAPbI_3_–DMTH, MAPbI_3_–Boc-PDA and MAPbI_3_–Tp throughout.

### 2.4. Preparation of MAPbI_3_ and COF–MAPbI_3_ Solutions

MAPbI_3_ precursor solutions were prepared outside the glovebox under ambient conditions at approximately 20% relative humidity. For pristine MAPbI_3_, MAI (0.19 g, 1.195 mmol, 0.92 equiv) and PbI_2_ (0.60 g, 1.302 mmol, 1.00 equiv) were dissolved in a mixed solvent of DMF (0.8 mL) and DMSO (0.2 mL), giving nominal concentrations of 1.20 M and 1.30 M, respectively, and stirred at 800 rpm and 60 °C for 2 h to obtain a clear solution. For COF-doped MAPbI_3_, the COF (0.5 mg, corresponding to 0.5 mg mL^−1^ or 0.063 wt% of the total solute mass) was dispersed in 1.0 mL of the MAPbI_3_ precursor solution by sonication for 30 min to yield a homogeneous ink. The dispersion was then stirred at 800 rpm and 60 °C for 2 h and passed through a 0.45 μm PTFE filter prior to deposition.

Perovskite films were deposited onto the m-TiO_2_/FTO substrates by a two-step spin-coating protocol. The COF–MAPbI_3_ ink was first spin-coated at 3000 rpm for 30 s. The spinning speed was then briefly reduced to 500 rpm for 2 s, during which 0.5 mL of chlorobenzene (antisolvent) was quickly dropped onto the rotating substrate. The speed was subsequently increased again to 3000 rpm for 30 s to remove excess solvent and promote rapid crystallization. The resulting films were annealed at 110 °C for 25 min to ensure complete conversion to MAPbI_3_. Control MAPbI_3_ films (without COF) were prepared using the same protocol but without COF addition. The final film thickness was ~500 nm, as determined by profilometry. The controlled incorporation of COFs into the MAPbI_3_ precursor at low loading enables us to probe how COF scaffolding influences nucleation, grain coalescence, and defect formation without significantly altering the bulk composition of the perovskite.

### 2.5. Device Fabrication

The resulting mesoscopic PSCs with the architecture FTO/c-TiO_2_/m-TiO_2_/perovskite/Spiro-OMeTAD/Au were fabricated. The hole-transporting layer (HTL) solution was prepared by dissolving Spiro-OMeTAD (72.4 mg) in chlorobenzene (1 mL), followed by the addition of 4-tert-butylpyridine (TBP, 28.8 μL) and a LiTFSI solution (17.3 μL of 450 mg mL^−1^ LiTFSI in acetonitrile). This HTL solution was spin-coated onto the COF:MAPbI_3_ (or pristine MAPbI_3_) layer at 3500 rpm for 30 s and dried at 60 °C for 15 min. Finally, a 100 nm Au top electrode was thermally evaporated through a shadow mask at a deposition rate of 0.3–0.5 Å s^−1^ under a base pressure of ~1 × 10^−6^ Torr. The active area of each device was 0.50 cm^2^, defined by the overlap of the FTO and Au electrodes.

### 2.6. Materials and Device Characterization

The crystal structure of the perovskite films was analyzed using a Proto A-XRD diffractometer equipped with a Cu Kα source (λ = 1.5406 Å) and a scintillation counter detector. Instrumental broadening was not subtracted from the measured full widths at half maximum. The crystallite sizes reported in Table 1 therefore represent apparent values and constitute lower bounds on the true coherent domain size. Since all films were measured on the same instrument under identical optical configuration, the relative comparison between samples is unaffected. UV–Vis absorption spectra of perovskite films on FTO were recorded with a Shimadzu UV-2550 spectrophotometer. Surface morphology, elemental composition, and elemental mapping were examined using a Zeiss field-emission scanning electron microscope (FE-SEM) operated at 20 kV. Film thicknesses were measured with a KLA-Tencor surface profilometer. Steady-state photoluminescence (PL) spectra were collected on a Horiba Fluorolog spectrometer equipped with a 532 W xenon lamp. Raman spectra were recorded using a Renishaw inVia spectrometer (532 nm excitation). Fourier-transform infrared (FTIR) spectra were recorded using a PerkinElmer Spectrum 400 spectrometer equipped with a diamond attenuated total reflectance (ATR) accessory. A background spectrum of the clean, empty ATR crystal was collected before sample measurement and subtracted automatically by the instrument software. No post-acquisition atmospheric compensation for water vapor or CO_2_ was applied. External quantum efficiency (EQE) measurements were performed using an Enli Technology QE-R system. Current density–voltage characteristics were recorded using a Keithley 2400 source meter under simulated AM1.5G illumination at 100 mW cm^−2^. Both scan directions were measured in a single continuous sweep cycle over the range −0.24 to 1.19 V at a scan rate of 0.286 V s^−1^ with no settling delay between the voltage step and the current measurement, and identical conditions were applied to all devices. Measurements were performed through a metal aperture mask defining the active area. The hysteresis index was calculated as HI = (PCE reverse − PCE forward)/PCE reverse.

## 3. Results and Discussion

**Figure 3:**
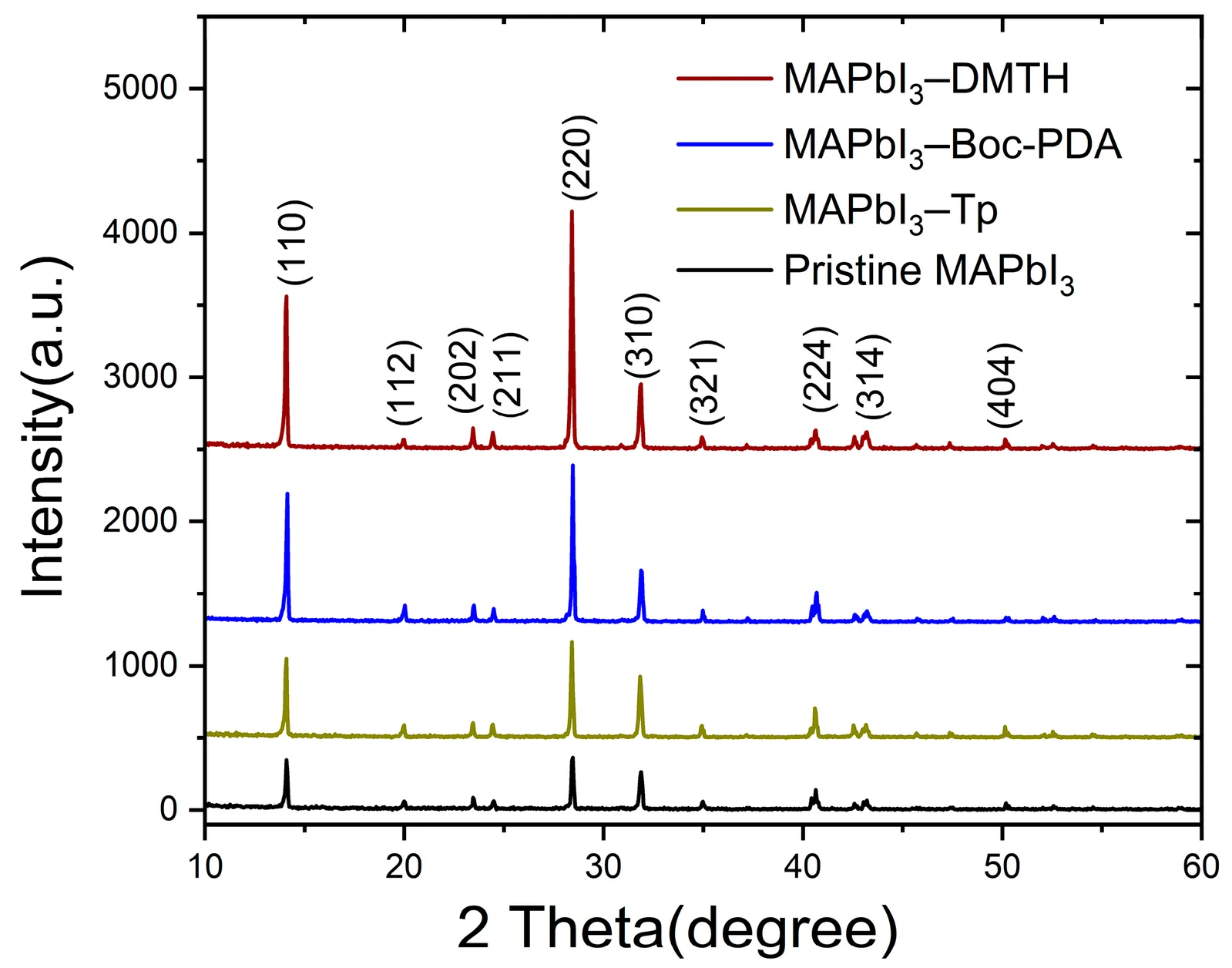
X-ray diffraction (XRD) patterns of pristine MAPbI_3_ and MAPbI_3_ films doped with different COFs: DMTH-COF, Boc-PDA-COF, and Tp-COF.

X-ray diffraction (XRD) analysis was employed to elucidate the crystallographic structure, phase purity, and microstructural evolution of MAPbI_3_ thin films in the presence of different COF additives. Figure 3 compares the diffraction patterns of pristine MAPbI_3_ with those of MAPbI_3_ films doped with low concentrations of COFs derived from DMTH, Boc-PDA, and Tp. All samples exhibit a consistent set of well-defined reflections at 2θ ≈ 14.26°, 20.04°, 22.95°, 24.53°, 28.04°, 31.92°, 34.05°, 40.24°, 43.12°, and 50.13°, which can be indexed to the (110), (112), (202), (211), (220), (310), (321), (224), (314), and (404) planes of the tetragonal MAPbI_3_ phase (ICDD 01-084-2960) [40,41]. The absence of additional peaks, particularly the PbI_2_ (001) reflection at ~12.7° or any broad amorphous features, confirms that the incorporation of COFs does not generate detectable secondary phases or disrupt the perovskite framework within the resolution of our measurements. This is a critical result: it demonstrates that the COF additives, at the concentrations used here, are structurally compatible with MAPbI_3_ and can be used to manipulate crystallization without sacrificing phase purity. Beyond phase identification, the XRD patterns provide quantitative insight into how COF chemistry influences the crystallization kinetics, crystalline coherence, and lattice strain of MAPbI_3_.

The lattice parameters, the crystallite size (D) calculated from the (220) peak, and microstrain (ϵ), of perovskite films were estimated using the following equation reported elsewhere [42,43,44]:

(1) D = 0.9λ / (β cos θ)

(2) ε = β / (4 tan θ)

(3) d = λ / (2 sin θ)

(4) 1 / d^2^ = (h^2^ + k^2^) / a^2^ + l^2^ / c^2^

where λ is the X-ray wavelength (0.15406 nm), θ is the Bragg angle (in radians; here evaluated near θ ≈ 28.5°), β is the FWHM (in radians) of the chosen diffraction peak, and (hkl) are the Miller indices corresponding to the tetragonal lattice parameters a and c. The quantity D obtained from Equation 1 is the coherent scattering domain size, that is, the length scale over which the lattice diffracts as a single periodic unit and is distinct from the physical grain size determined below by FE-SEM. Values of D are uncorrected for instrumental broadening. D and ε are estimated here under the limiting assumptions of size-only and strain-only broadening, respectively, and are therefore indicative parameters rather than a full deconvolution of the two contributions. The extracted lattice parameters, FWHM values, crystallite sizes, and microstrain for all samples are summarized in Table 1.

The lattice parameters remain essentially invariant across all samples, with (a ≈ 8.895) Å and (c ≈ 12.758) Å, confirming that the introduction of Tp, Boc-PDA, or DMTH does not alter the average tetragonal MAPbI_3_ crystal structure. By contrast, the FWHM, crystallite size (D), and microstrain ε show a clear and systematic dependence on COF chemistry. Pristine MAPbI_3_ exhibits a relatively broad FWHM of 0.23°, corresponding to an average crystallite size of 35.6 nm and a microstrain of 3.56 × 10^−3^. Upon incorporation of Tp-COF, the FWHM decreases to 0.18°, and the crystallite size increases to 45.49 nm, accompanied by a reduction in microstrain to 2.76 × 10^−3^. Boc-PDA-COF further improves these parameters, yielding (D = 48.17) nm and ε = 2.53 × 10^−3^ (FWHM = 0.17°). The most pronounced effect is observed for the DMTH-modified film, which displays the narrowest peak (FWHM = 0.15°), the largest crystallite size (54.59 nm), and the lowest microstrain (2.26 × 10^−3^). The crystallite sizes obtained here, 35.6–54.6 nm, are an order of magnitude smaller than the mean grain sizes of 0.36–1.00 μm determined by FE-SEM in the following section, as expected since each grain accommodates multiple coherently diffracting domains. Both quantities increase in the same order across the additive series, indicating that the COFs enlarge the grains and improve the crystalline coherence within them at the same time.

This systematic evolution, characterized by larger crystallite size (D) and smaller microstrain (ε) from pristine MAPbI_3_ to Tp-, Boc-PDA-, and finally DMTH-modified films, provides strong quantitative evidence that COF chemistry governs MAPbI_3_ crystallization kinetics and defect formation. The Tp-based system already acts as an effective scaffold, promoting more coherent crystalline growth and partially relaxing lattice distortion. Introducing Boc-PDA further enriches the framework with amino functionality, enhancing interactions with PbI_2_/MAI species and leading to a further increase in crystallite size and strain relief. DMTH-COF, which combines methoxy-substituted aromatic rings with hydrazide groups, exerts the strongest effect: its richer coordination and hydrogen-bonding environment most effectively regulates nucleation and supports the growth of larger, more ordered perovskite domains with minimal residual strain. The XRD and extracted microstructural parameters show that all three COFs preserve the tetragonal MAPbI_3_ phase while progressively sharpening the diffraction peaks, enlarging the crystallites, and reducing microstrain. Among them, DMTH-COF stands out as the most efficient crystallization regulator, delivering the largest coherent domain size and the lowest lattice distortion. This structural optimization, namely phase-pure tetragonal MAPbI_3_ with enlarged grains and reduced microstrain, provides the crystallographic foundation for the improved photophysical properties and device performance observed in the subsequent PL and photovoltaic measurements [45].

**Figure 4:**
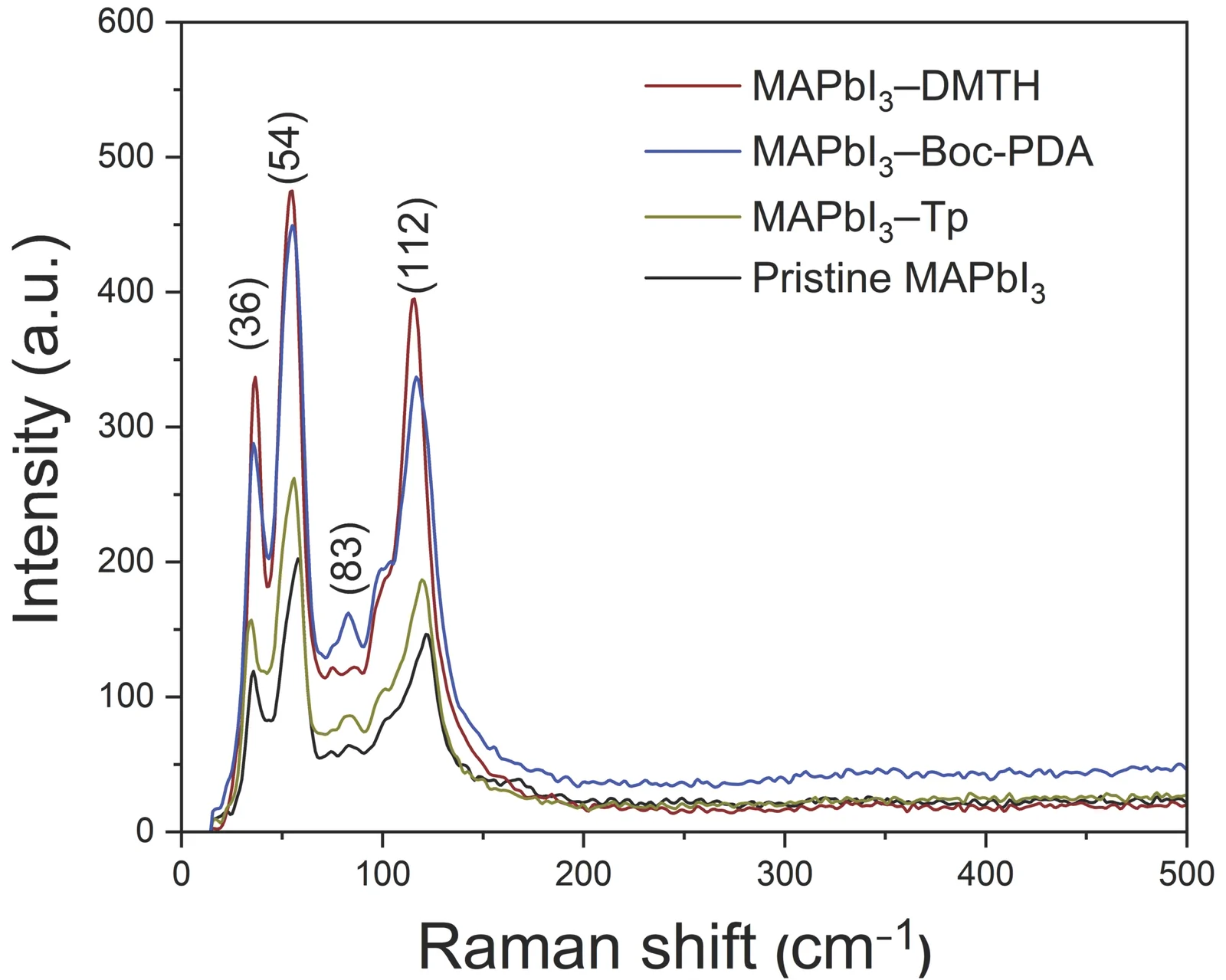
Raman spectra of pristine MAPbI_3_ and COF-modified MAPbI_3_ thin films recorded with 532 nm excitation, showing the low-wavenumber region dominated by lattice vibrational modes.

Raman spectroscopy was employed to probe how COF additives affect the molecular and lattice dynamics of MAPbI_3_, complementing the XRD analysis of long-range order. Spectra were collected using a confocal Raman microscope with a 532 nm laser (200 mW) in the 0–500 cm^−1^ range, which captures the key inorganic framework vibrations and MA^+^-related modes while avoiding strong substrate contributions. Figure 4 compares the Raman response of pristine MAPbI_3_ with that of films prepared with DMTH, Boc-PDA, and Tp-based COFs. In all cases, the spectra exhibit characteristic low-wavenumber bands that are consistent with the tetragonal MAPbI_3_ phase reported in the literature [46,47]. The Raman spectra are dominated in the low-wavenumber region by distinct features at approximately 36, 54, 83, and 112 cm^−1^ [48,49,50]. These bands arise mainly from Pb–I stretching and bending vibrations within the corner-sharing [PbI_6_]^4−^ octahedral framework, together with lattice modes involving coupled displacements of the MA^+^ cations [48]. The preservation of these sharp, phase-specific vibrational signatures, together with the absence of additional peaks associated with PbI_2_ or other degradation products, is fully consistent with the XRD results and confirms that all COF-treated films retain the tetragonal MAPbI_3_ perovskite structure. Although the overall vibrational fingerprint of MAPbI_3_ is preserved, careful inspection reveals systematic COF-induced modifications in both peak position and line shape.

In particular, the Pb–I lattice modes shift progressively toward lower wavenumbers in the COF-treated films. To quantify this, each band was fitted with a Lorentzian profile over an identical spectral window for all four samples, and the extracted positions are given in Table 2.

Lorentzian fitting was performed to quantify the positions of the selected low-wavenumber Raman bands (Table 2). The lower-frequency mode was located at 58.22 cm^−1^ for pristine MAPbI_3_ and shifted to 55.97, 55.44, and 54.71 cm^−1^ after modification with Tp, Boc-PDA, and DMTH, respectively. The corresponding red shifts were 2.25, 2.78, and 3.51 cm^−1^. Similarly, the higher-frequency mode shifted from 121.88 cm^−1^ for the pristine film to 119.93, 118.17, and 115.88 cm^−1^ for the Tp-, Boc-PDA-, and DMTH-modified films, respectively. These shifts are consistent with modification of the local Pb–I vibrational environment and may reflect changes in lattice strain, bonding interactions, or the electrostatic environment surrounding the PbI_6_ octahedra [47]. Their magnitudes follow the same ordering as the crystallite size and microstrain from XRD and the grain size from FE-SEM, providing independent vibrational evidence for the progressive relaxation of the Pb–I framework.

The relative changes among the three COFs are particularly revealing. The 36 and 83 cm^−1^ features were not analyzed quantitatively; the former, because it does not follow a consistent trend across the series and the latter because its low intensity and overlap with the 112 cm^−1^ mode preclude a reliable fit. The Tp-modified film already exhibits a modest narrowing and slight intensity enhancement of the lattice modes compared to the pristine reference, suggesting that even a relatively rigid, less functionalized COF can improve vibrational coherence. The Boc-PDA-COF system further sharpens the Pb–I features and strengthens the modes associated with MA^+^ libration, which is consistent with amino-containing linkers interacting more directly with the organic cations [51]. The DMTH-containing films show the most pronounced effects, with the largest narrowing and the strongest intensity increase in the mixed Pb–I–MA region. This behavior can be attributed to the richer hydrogen-bonding and coordination landscape provided by the methoxy and hydrazide groups in DMTH-COF, which are well suited to interact simultaneously with the inorganic framework and the MA^+^ cations [52]. These interactions likely damp large-amplitude MA^+^ reorientations and reduce dynamic disorder at grain boundaries, leading to a more coherent vibrational response.

The Raman data provide molecular-level support for the proposed COF-assisted crystallization mechanism. The preservation of phase-specific modes indicates that the perovskite lattice is not disrupted by COF incorporation. The red shifts and peak sharpening demonstrate that COFs relieve local strain and suppress vibrational disorder, in agreement with the larger crystallite sizes and lower microstrain extracted from XRD. Among the three systems, DMTH-COF stands out as the most effective modifier of the vibrational landscape, which aligns with its superior impact on grain growth and defect suppression. These spectroscopic signatures strongly suggest that COFs act as static structural templates during film formation and also as dynamic modulators of lattice and cation motions. This dual role is highly relevant for device performance, since reduced vibrational disorder and stabilized MA^+^ dynamics are expected to lower non-radiative recombination, hinder ion migration, and ultimately contribute to the enhanced photostability and efficiency observed in the corresponding solar cells [53].

**Figure 5:**
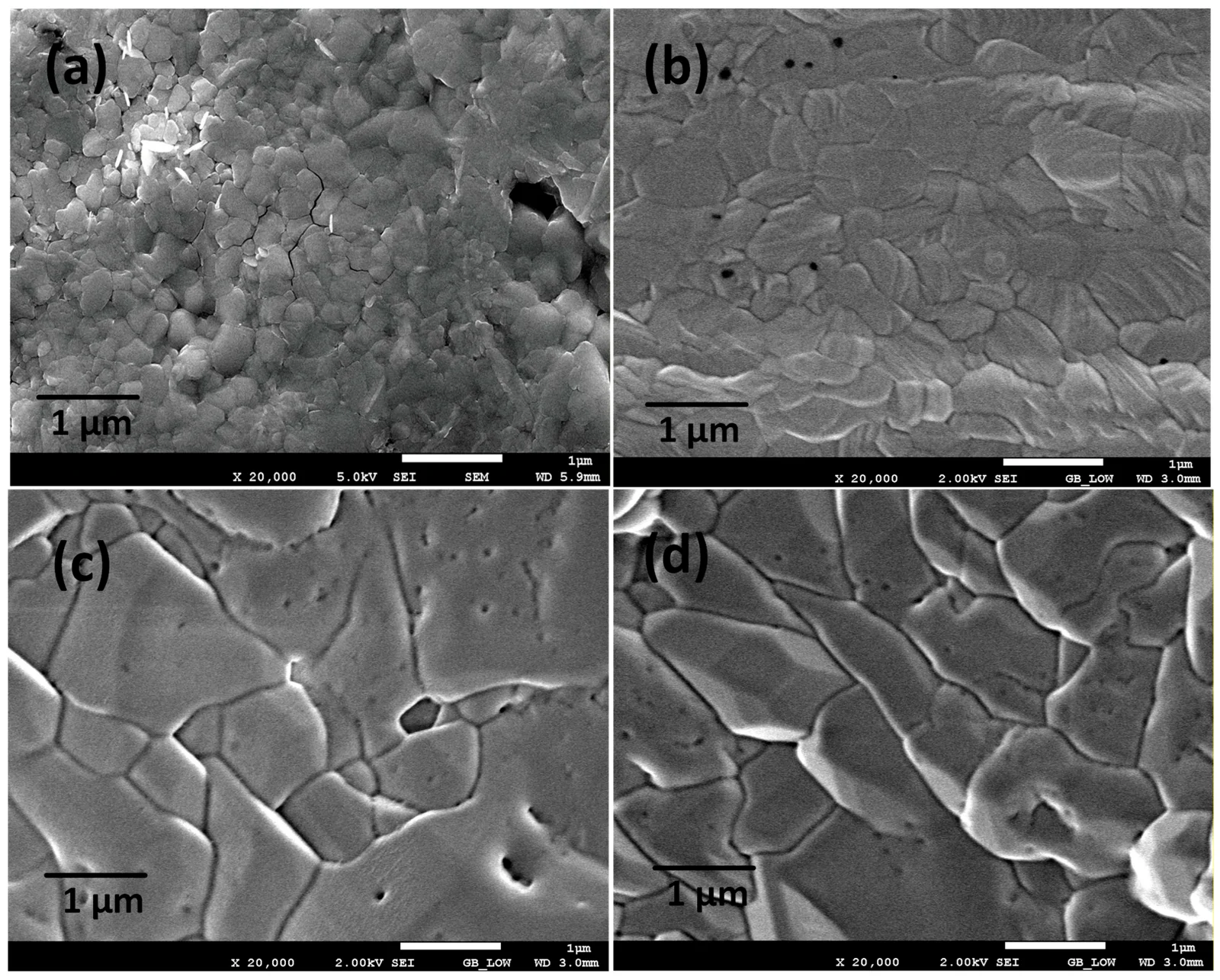
Top-view FE-SEM images of (**a**) pristine MAPbI_3_ and MAPbI_3_ thin films doped with (**b**) Tp-COF, (**c**) Boc-PDA-COF, and (**d**) DMTH-COF. All films were deposited by spin-coating on TiO_2_-based substrates.

To elucidate how COF incorporation modifies the microstructure of MAPbI_3_, we examined the surface morphology of the perovskite films by field-emission scanning electron microscopy, as shown in Figure 5. All samples were prepared by spin-coating on TiO_2_-based substrates under identical conditions, with low loadings of COFs chosen to promote crystallization while preserving the underlying perovskite lattice. The COFs studied here, DMTH, Boc-PDA, and Tp, are relatively under-explored in the context of perovskite thin films, which allows us to directly assess their potential as morphology-directing additives. The pristine MAPbI_3_ film (Figure 5a) exhibits a compact but fine-grained morphology, with grains averaging around 360 nm. Although the surface coverage is reasonably uniform, the high density of grain boundaries and the presence of small voids and irregular grain shapes are evident. Such features are typically associated with elevated densities of grain-boundary defects, which can act as non-radiative recombination centers and scattering sites, limiting carrier mobility and diffusion length in photovoltaic devices [54].

Upon incorporation of COF additives, the morphology changes dramatically. The Tp-doped film (Figure 5b) shows grains that have grown to roughly 670 nm on average, with improved grain coalescence and fewer visible intergranular gaps. Boc-PDA-COF further accentuates this trend (Figure 5c), producing grains of about 910 nm, as well as a noticeably smoother and more continuous surface. The most pronounced effect is observed for DMTH-COF (Figure 5d), where the average grain size reaches about 1 µm, corresponding to more than a threefold increase compared to the pristine film. In this case, the surface is dominated by large, faceted grains with well-defined boundaries, and the film appears nearly pinhole-free at the micron scale. These systematic morphological changes indicate that the COFs strongly influence the nucleation and growth kinetics of MAPbI_3_. The enlarged grains and reduced boundary density suggest that COFs act as crystallization regulators or soft templates during film formation [55]. Mechanistically, this behavior can be rationalized by the functional groups and porous architectures intrinsic to these COFs. The hydrazide and imine (C=N) functionalities provide coordination sites for Pb^2+^ species, while amino and methoxy groups can form hydrogen bonds with MA^+^ cations or halide anions [56,57]. Such interactions are expected to moderate the rate of nucleation, promote the growth of fewer but larger nuclei, and stabilize the evolving grain boundaries [58]. At the same time, the porous and π-conjugated networks of the COFs likely facilitate more uniform solvent evaporation and enhance mass transport during the crystallization step, which further supports the formation of large, well-connected grains [59].

Incorporation of COFs therefore affects the geometric microstructure and the defect landscape. Fewer grain boundaries and smoother interfaces imply a lower density of undercoordinated Pb^2+^ and halide vacancies at the surface and interfaces [60]. The coordination bonding between COF C=N groups and Pb^2+^ ions, coupled with hydrogen bonding between COF −NH_2_ groups and halides, can effectively passivate both shallow and deep traps at and near grain boundaries [61]. This structural passivation is consistent with the observed narrowing of XRD peaks and reduced microstrain. From a device standpoint, the grain enlargement and improved surface quality induced by COF additives are expected to have several beneficial consequences. Larger grains and fewer boundaries reduce non-radiative recombination pathways, allowing photogenerated carriers to travel longer distances before recombination, which should enhance charge collection and increase open-circuit voltage [62]. Better grain connectivity and reduced porosity facilitate more efficient charge transport across the perovskite layer, which can translate into higher fill factors and improved device performance [63]. Additionally, the partial hydrophobic character introduced by certain COF moieties, particularly in the DMTH-modified films, may help to improve environmental stability by limiting moisture ingress along grain boundaries [64]. Importantly, the use of DMTH, Boc-PDA, and Tp as direct dopants inside the perovskite layer represents a distinct approach compared with most previous studies, which have concentrated on more conventional imine-linked COFs used mainly at interfaces or as surface passivation layers [65,66]. Here, the COFs actively guide bulk film formation and microstructure rather than only decorating the surface. The FE-SEM results thus provide direct visual evidence that COF chemistry can be harnessed to engineer the grain-scale architecture of MAPbI_3_, laying a structural foundation for the improved optoelectronic properties and photovoltaic performance that will be discussed in the following sections.

**Figure 6:**
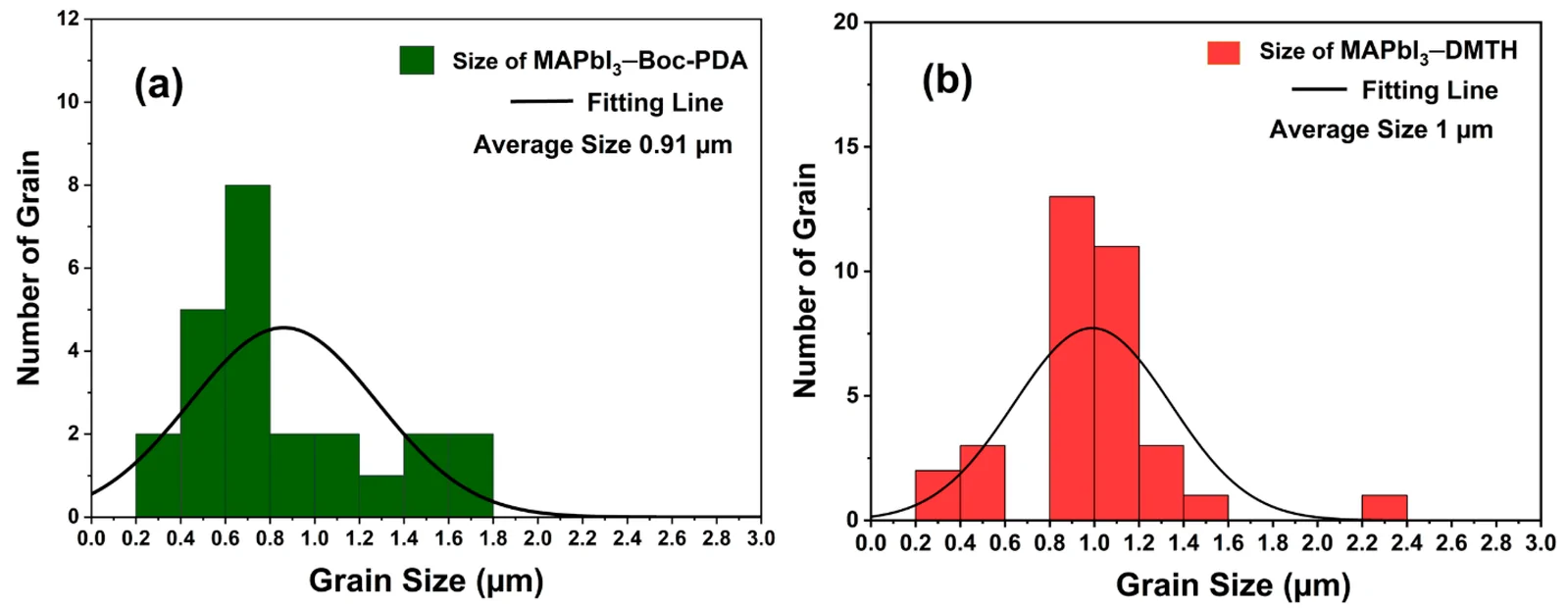
Grain size distribution histograms for MAPbI_3_ perovskite thin films doped with (**a**) Boc-PDA-COF and (**b**) DMTH-COF, obtained from statistical analysis of plan-view FE-SEM images. The mean grain sizes are approximately 0.91 μm for the Boc-PDA-COF film and 1.0 μm for the DMTH-COF film. In both cases, the experimental grain size distributions are well fitted by lognormal functions (solid curves), reflecting the skewed but unimodal grain populations characteristic of COF-regulated solution crystallization.

Figure 6 shows the grain size distributions of COF-modified MAPbI_3_ films and quantitatively confirms the strong grain-growth effect inferred from the FE-SEM images. The histograms were obtained by measuring individual grains from plan-view FE-SEM micrographs and plotting the frequency of occurrence as a function of equivalent grain diameter for MAPbI_3_ films doped with (a) Boc-PDA-COF and (b) DMTH-COF. In both cases, the distributions exhibit the characteristic positive skew typical of solution-processed perovskites and are well described by lognormal fits, indicating that nucleation and growth follow a multiplicative process. The Boc-PDA-COF film displays a mean grain size of approximately 0.91 μm, with the majority of grains clustered around 0.6–0.8 μm and relatively few sub-500 nm crystallites, indicating efficient suppression of excessive nucleation and enhanced grain coalescence. The DMTH-COF film shows an even more pronounced shift toward larger grains, with a mean diameter near 1.0 μm and a distribution that is both broadened toward larger sizes and depleted of small grains. This evolution of the lognormal parameters between Boc-PDA-COF and DMTH-COF provides strong evidence that COF chemistry systematically modulates the statistics of MAPbI_3_ grain growth: DMTH-COF, with its more strongly coordinating hydrazide and methoxy functionalities, promotes fewer nucleation events and more extensive lateral growth per nucleus, yielding a microstructure dominated by large, well-connected grains and a significantly reduced grain-boundary density.

**Figure 7:**
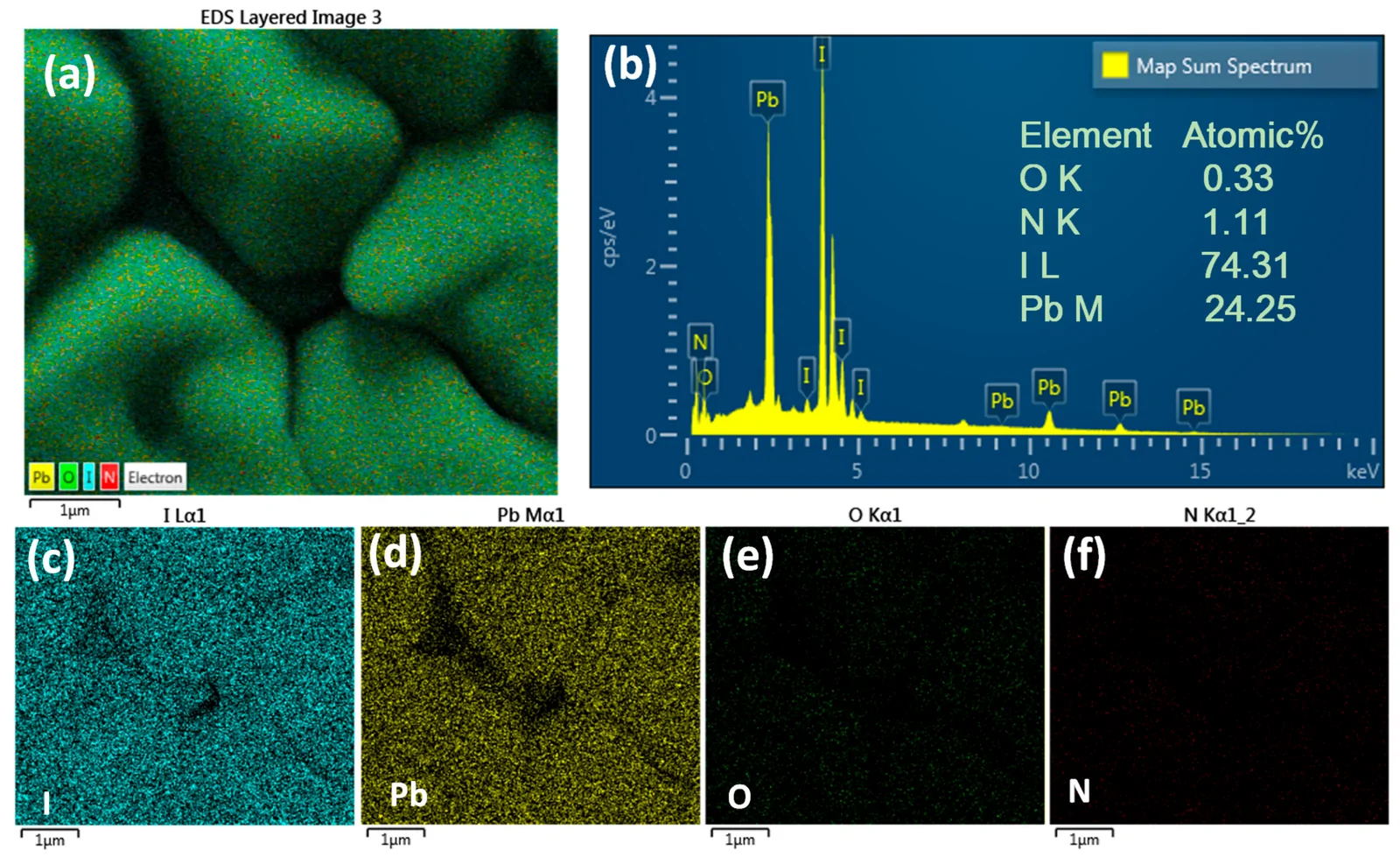
Energy-dispersive X-ray spectroscopy of the DMTH-COF-modified MAPbI_3_ film. (**a**) Layered elemental map showing the combined Pb, O, I and N signals overlaid on the secondary electron image. (**b**) Summed EDX spectrum with the quantified atomic fractions, giving a Pb:I ratio of 1:3.06. (**c**–**f**) Individual elemental maps for I Lα, Pb Mα, O Kα and N Kα, respectively. All scale bars are 1 μm.

To evaluate the elemental composition and spatial distribution within the DMTH-COF-modified MAPbI_3_ film, we conducted energy-dispersive X-ray spectroscopy (EDX). The summed spectrum (Figure 7b) is dominated by characteristic iodine and lead emissions, yielding atomic fractions of 74.31% I and 24.25% Pb. The resulting Pb:I ratio of 1:3.06 aligns closely with the nominal 1:3 stoichiometry of MAPbI_3_, confirming that the COF additive does not disrupt the bulk inorganic framework [67]. This compositional stability is critical: deviations from ideal stoichiometry typically generate halide vacancies or metallic lead clusters, both of which act as deep trap states and exacerbate non-radiative recombination. Consequently, the enhanced carrier lifetime and device performance cannot be attributed to compositional shifts in the bulk perovskite, but rather to the structural and interfacial improvements identified via our diffraction and morphological analyses. The elemental maps further demonstrate that this near-ideal stoichiometry is uniform across the film’s microstructure. Both iodine and lead signals (Figure 7c,d) are distributed continuously across the grain interiors and boundaries. We observe no regions of halide depletion or lead enrichment, which would indicate halide segregation or PbI_2_ accumulation at the grain edges.

The absence of such segregation is consistent with the XRD data, where no residual PbI_2_ reflection was detected at 12.7°, confirming that the enlarged grains induced by the COF remain compositionally homogeneous. Signals for nitrogen (1.11 at.%) and oxygen (0.33 at.%) were also detected, and their corresponding maps (Figure 7e,f) show a uniform distribution across the imaged area. Because light elements are notoriously difficult to quantify accurately by EDX due to severe self-absorption and low detector efficiency at the low energies of the N Kα (0.392 keV) and O Kα (0.525 keV) lines, these absolute values should be treated as qualitative. However, the spatial mapping is highly informative: the lack of localized N or O accumulation confirms that the additive does not aggregate into discrete, phase-separated organic domains. Instead, the COF remains homogeneously dispersed throughout the film. Together, these results indicate that the additive facilitates uniform crystallization and surface passivation without perturbing the phase purity or bulk composition of the perovskite absorber.

Water contact angle measurements were performed to quantitatively examine how COF doping modifies the surface wettability and, by implication, the surface energy and moisture affinity of MAPbI_3_ thin films. Because MAPbI_3_ degradation is strongly accelerated by humidity and frequently initiates at exposed surfaces and grain boundaries [68], even relatively small changes in surface hydrophilicity or hydrophobicity can have a disproportionate impact on long-term stability and interfacial reliability in devices [69]. In this work, three COF-type organic additives based on DMTH, Boc-PDA, and Tp were incorporated into MAPbI_3_, and their influence on WCA was systematically compared with that of undoped MAPbI_3_. The WCA analysis therefore provides a direct, macroscopic probe of how COFs alter the outermost surface chemistry and morphology, linking microstructural changes observed by FE-SEM and XRD to the improvements in device performance and stability.

**Figure 8:**
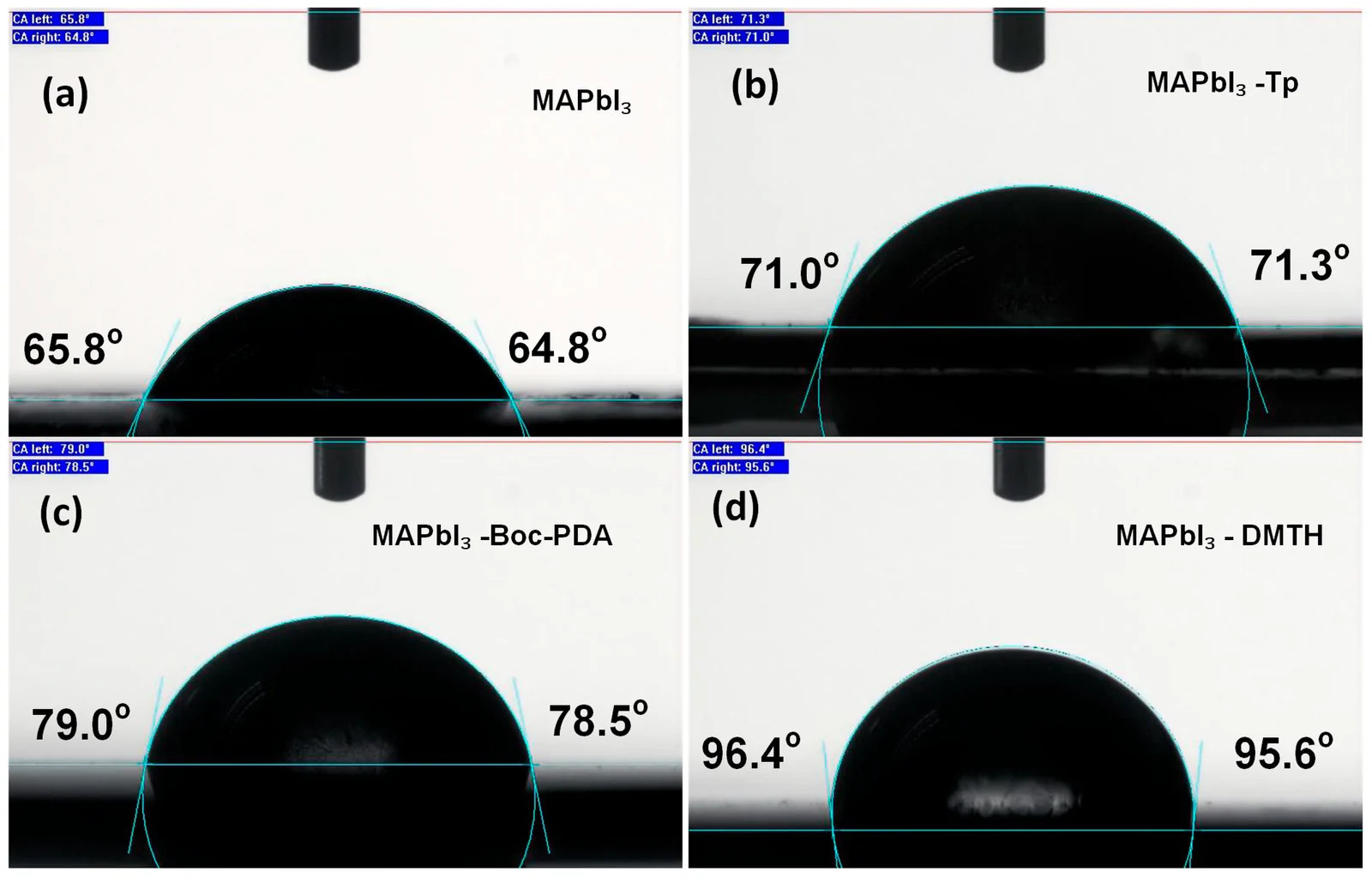
Water contact angle (WCA) images for (**a**) pristine MAPbI_3_, (**b**) MAPbI_3_–Tp, (**c**) MAPbI_3_–Boc-PDA, and (**d**) MAPbI_3_–DMTH films, measured by the sessile-drop method. Left and right contact angles of each droplet (shown in the images) were averaged to obtain the WCA values reported in the text.

All WCA measurements were carried out using the sessile-drop method on a contact angle goniometer. MAPbI_3_ and MAPbI_3_–COF films (MAPbI_3_–Tp, MAPbI_3_–Boc-PDA, and MAPbI_3_–DMTH) were deposited on identically cleaned TiO_2_/FTO substrates, employing the same precursor concentration, spin parameters, antisolvent timing, and annealing protocol described in the experimental section. A microsyringe was used to gently place small droplets (4 µL) of deionized water onto the film surface without mechanically disturbing the perovskite layer. Representative droplet profiles are shown in Figure 8. The pristine MAPbI_3_ film (Figure 8a) exhibits a moderate an average contact angle of 65.3°, corresponding to partial wetting and a surface that is neither strongly hydrophilic nor strongly hydrophobic. This behavior is consistent with the mixed ionic and organic nature of MAPbI_3_ and the presence of polar methylammonium cations and undercoordinated ions at the surface [70]. While such an intermediate WCA favors wetting by typical solution-processed transport layers, it also leaves the surface vulnerable to water adsorption from ambient air, which can initiate hydrolysis and decomposition [71]. This pristine WCA therefore serves as a meaningful baseline for assessing the effect of COF incorporation.

When Tp-based COF is introduced (Figure 8b, MAPbI_3_–Tp), the water droplet becomes slightly more spherical, and the contact angle increases to about 71.1°. This change reflects a modification of the surface free energy, likely due to the presence of aromatic rings and keto/enolic groups in the Tp-derived network that partially shield polar sites at the perovskite surface or at grain boundaries [72]. Even this modest increase suggests that the COF is not confined to the film interior but influences the near-surface region, either through surface segregation or preferential decoration of grain boundaries that intersect the surface. A more pronounced effect is seen for the film doped with Boc-PDA-derived COF (Figure 8c, MAPbI_3_–Boc-PDA), where the WCA rises to approximately 78.7°. The Boc-PDA-COF motif introduces bulky aromatic and urethane-like groups that can reorient at the film–air interface and alter local packing [73]. The higher WCA indicates a more hydrophobic surface, which reduces direct contact between water molecules and polar perovskite sites. Combined with FE-SEM evidence of smoother, denser morphologies and XRD indications of improved crystallinity, this increase in WCA supports the interpretation that Boc-PDA-based COF effectively passivates surface defects and grain boundaries, thereby limiting moisture access to reactive sites and contributing to enhanced stability.

The most dramatic change in wettability is observed for the DMTH-containing film (Figure 8d, MAPbI_3_–DMTH), where the WCA reaches approximately 96°. The significantly more spherical droplet profile reveals a highly hydrophobic surface compared to the pristine and other COF-modified films. Given the functional architecture of DMTH-COF, which combines methoxy and hydrazide groups within a conjugated framework, this strong hydrophobic response can be attributed to a combination of topographical smoothing, partial coverage of hydrophilic perovskite sites by the COF, and reorientation of less polar COF moieties toward the outer surface [74]. The concomitant microstructural improvements seen in FE-SEM for DMTH-doped films, including large, well-coalesced grains with minimal pinholes, further reinforce the view that DMTH-COF not only regulates bulk crystallization but also significantly reorganizes the surface region. The series of WCA images and corresponding values reveal a clear, monotonic tuning of surface wettability with COF chemistry. Relative to pristine MAPbI_3_, all three COF-doped films exhibit higher contact angles, confirming that COF addition is an effective strategy for increasing the hydrophobic character of the perovskite surface. This trend is particularly pronounced for DMTH-COF, which yields a hydrophobic surface regime. When these results are considered alongside FE-SEM (denser, larger grains), XRD (enlarged crystallites and reduced microstrain), PL (enhanced radiative recombination and reduced non-radiative loss), and device performance data (higher Jsc, FF, PCE, together with improved operational stability), a coherent picture emerges. COF additives simultaneously refine the bulk microstructure and engineer the outermost surface to be less accessible to moisture and other environmental stressors [75]. The WCA measurements therefore provide an essential mechanistic link between molecular-scale surface modification by COFs and the macroscopic gains in efficiency and durability observed in MAPbI_3_ perovskite solar cells.

**Figure 9:**
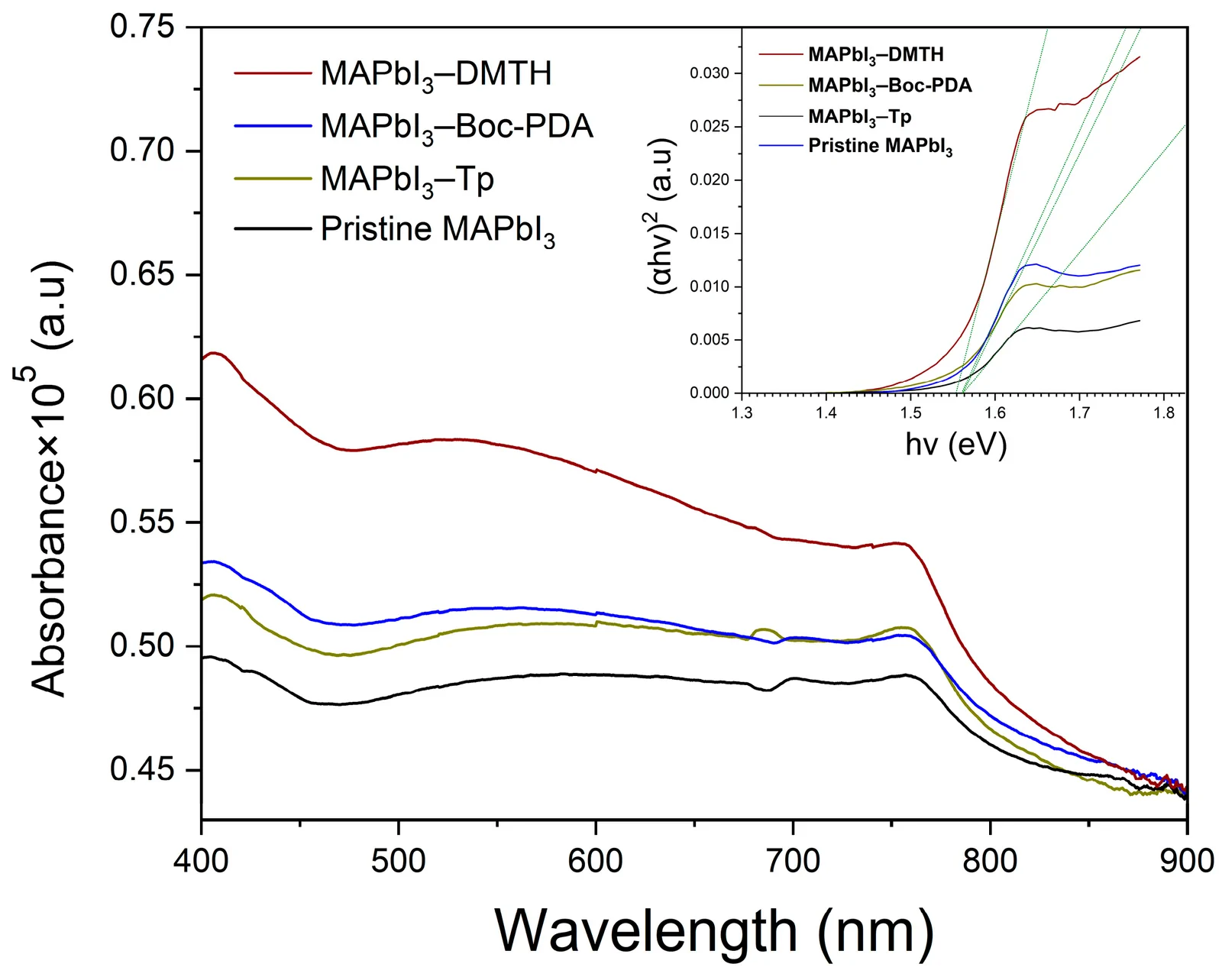
UV–Vis absorbance spectra of pristine MAPbI_3_ and MAPbI_3_ thin films modified with DMTH-COF, Boc-PDA-COF, and Tp-COF, recorded in the 400–900 nm range.

UV–Vis spectrophotometry was employed to elucidate how COF additives influence the optical response and band structure of MAPbI_3_ thin films. Absorbance spectra were recorded using a double-beam spectrophotometer over 400–900 nm with a 1 nm resolution. All films examined by UV–Vis spectroscopy were prepared using the same perovskite precursor concentration and an identical additive concentration of 0.5 mg mL^−1^, under the same deposition and annealing conditions, allowing direct comparison of their measured spectra. The four spectra coincide below the absorption edge and separate only above it, confirming that the differences in absorbance arise from the optical response of the films rather than from any difference in thickness or absorber loading. As shown in Figure 9, all spin-coated films exhibit strong absorption across the visible spectrum with a pronounced onset near 750–780 nm, consistent with the direct bandgap transition of MAPbI_3_ arising from electronic transitions between valence-band states derived primarily from I 5p and Pb 6s orbitals and conduction-band states dominated by Pb 6p orbitals [76,77]. The high absorbance in this region reflects the intrinsically large absorption coefficient of hybrid lead halide perovskites [78]. The pristine MAPbI_3_ film presents a relatively sharp absorption edge and smooth spectral profile. Upon incorporation of Tp, Boc-PDA, and DMTH-based COFs, the overall absorbance in the visible range increases while the absorption edge remains unchanged. Among the modified samples, the DMTH-containing film displays the highest absorbance over a broad wavelength range. This enhancement can be attributed to improved film coverage and crystallinity, as corroborated by FE-SEM and XRD, and to additional electronic interactions between the π-conjugated COF and the perovskite lattice that increase the effective oscillator strength and reduce scattering losses [79].

To quantify the impact on the optical bandgap, the absorption data were analyzed using Tauc plots for direct-bandgap semiconductors, according to

(5) (*αhν*)*^n^* = *A*(*hν* − *E_g_*)

where *α* = Absorption coefficient, *h* = Planck’s constant, *ν* = Frequency, *n* = Exponent related to the transition type, *A* = Constant, and *E*_g_ = Band gap energy. Extrapolation of the linear region of ((αhν)^2^) versus (hν) gives bandgap values of 1.567 eV for pristine MAPbI_3_, 1.567 eV for the Tp-doped film, 1.567 eV for the Boc-PDA-doped film and 1.560 eV for the DMTH-doped film, equal to within 7 meV (inset of Figure 9). The absorption onset is therefore unaffected by COF incorporation, as expected for additives too large to be accommodated on a lattice site, and consistent with the invariant tetragonal lattice parameters reported in Table 1.

These optical trends are significant for several reasons. First, the preservation of a sharp absorption onset and the absence of additional sub-bandgap features confirm that COF incorporation does not generate optically active defect phases, which is consistent with the phase-pure XRD patterns and clean vibrational signatures seen in the Raman data. Second, the increased absorbance across the visible range reflects improved film coverage and crystallinity, which enhances photon harvesting within the same spectral window and contributes to the higher short-circuit current density [80]. Third, the strongest effects are observed for the DMTH-modified film, which is consistent with its superior structural and vibrational characteristics. The methoxy and hydrazide functionalities in DMTH-COF are expected to donate electron density and form hydrogen bonds at the perovskite surface, stabilizing the lattice and slightly perturbing the band edges. The combination of higher absorbance, an unchanged bandgap and maintained spectral cleanliness reflects a favorable modification of the perovskite morphology without alteration of its electronic structure. These optical improvements, when considered together with the enlarged grain sizes, reduced microstrain, and more coherent lattice dynamics, point to a coherent picture in which COF-engineered MAPbI_3_ films are better suited to high-efficiency and potentially more stable perovskite solar cells, with improved visible-light absorption.

**Figure 10:**
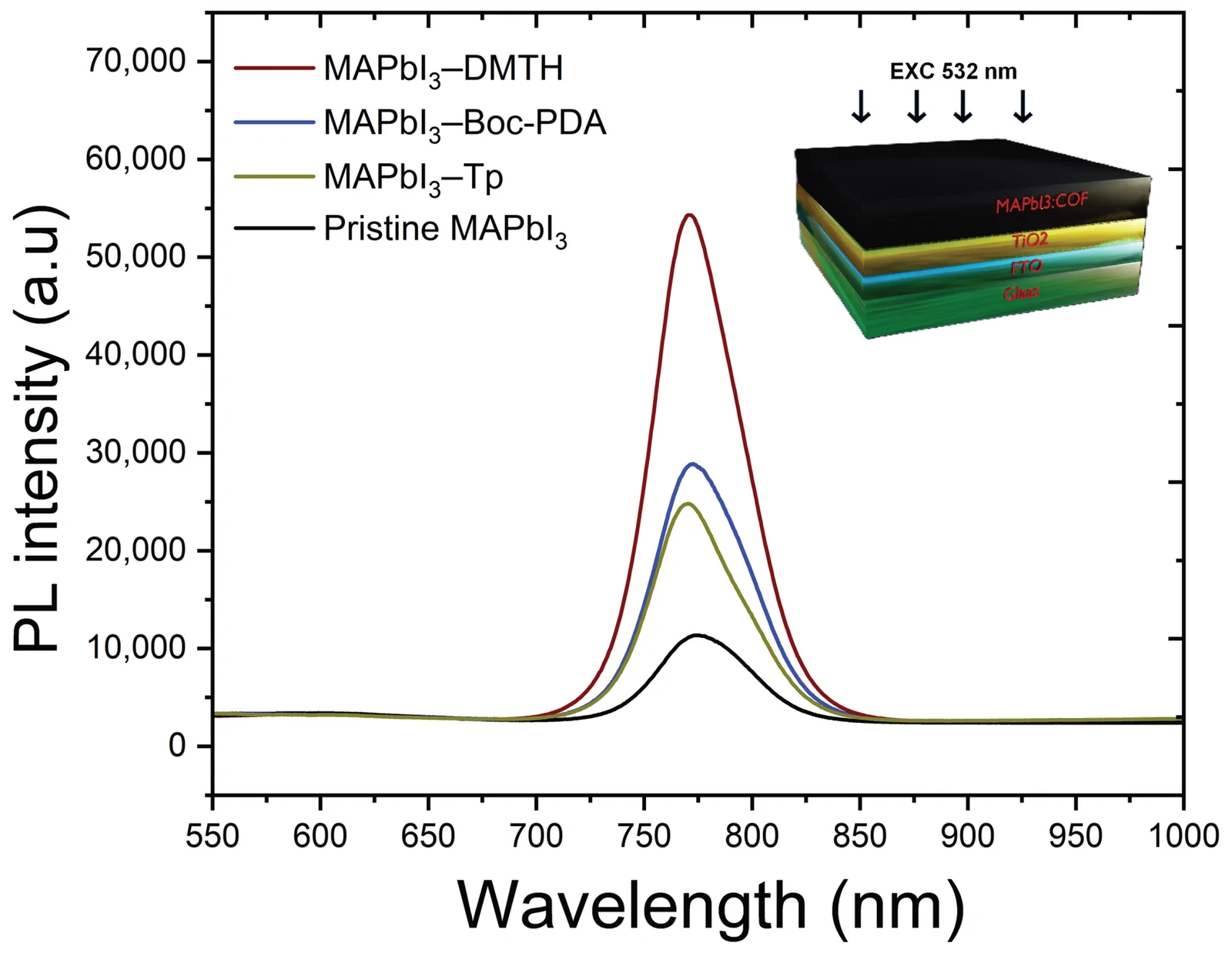
Steady-state photoluminescence (PL) spectra of pristine MAPbI_3_ and COF-modified MAPbI_3_ thin films (DMTH-COF, Boc-PDA-COF, Tp-COF) deposited on TiO_2_ substrates, recorded in the 550–1000 nm range under 532 nm excitation (80 mW cm^−2^).

Photoluminescence spectroscopy provides a direct probe of radiative and non-radiative recombination processes in perovskite absorbers and is therefore a sensitive indicator of defect density, trap passivation, and carrier dynamics. Here, steady-state PL measurements were performed on MAPbI_3_ films spin-coated onto compact/mesoporous TiO_2_ substrates, with emission collected between 550 and 1000 nm under 532 nm excitation. Figure 10 compares the PL response of pristine MAPbI_3_ with that of films incorporating DMTH, Boc-PDA, and Tp-based COFs. All three films were prepared at the same additive loading and film thickness and measured under identical excitation conditions, so the absorbed photon flux is comparable across the series. All samples display a dominant emission peak in the 750–800 nm region, consistent with band-edge recombination in tetragonal MAPbI_3_ [81]. All spectra exhibit a single, intense emission band centered around 772 nm (approximately 1.6 eV), in excellent agreement with the optical bandgap extracted from UV–Vis measurements.

The nearly invariant PL peak position therefore indicates that Tp, Boc-PDA, and DMTH do not substantially alter the fundamental bandgap of MAPbI_3_. Accordingly, the differences in the apparent Tauc bandgaps are interpreted as variations in the near-edge optical response rather than evidence of pronounced bandgap narrowing. The observed changes in PL intensity are more reasonably associated with changes in defect-mediated non-radiative recombination and film quality [82]. Furthermore, the absence of secondary peaks or shoulders and the nearly Gaussian line shape indicate minimal contributions from phase impurities, self-trapped excitons, or strong photon reabsorption [83]. This clean, unimodal profile is fully consistent with the phase-pure tetragonal MAPbI_3_ structure established by XRD and the well-defined vibrational fingerprints observed in Raman spectra. The most striking feature in Figure 10 is the systematic enhancement of PL intensity upon COF incorporation. Relative to pristine MAPbI_3_, all COF-treated films show significantly higher PL, with the strongest emission observed for the DMTH-modified film, followed by Boc-PDA-COF and then Tp-COF. Since the emission peak position remains essentially unchanged, this increase in PL intensity can be attributed primarily to a reduction in non-radiative recombination pathways [84]. The COF additives improve the internal radiative efficiency of the perovskite by passivating electronic defects that would otherwise act as non-radiative recombination centers. These spectroscopic indications align closely with the structural and morphological trends identified in the previous sections. Structural analysis revealed that COF-treated films possess larger crystallite sizes and lower microstrain than pristine MAPbI_3_, particularly in the DMTH-COF case. FE-SEM images showed more compact, well-coalesced grains with fewer pinholes for COF-modified films. Such structural improvements are expected to decrease the density of undercoordinated Pb^2+^ and halide vacancies at grain boundaries, which are known to act as deep traps [85]. The enhanced PL intensity is therefore consistent with a scenario in which COFs, and especially the hydrazide and methoxy functionalities in DMTH-COF, coordinate to undercoordinated Pb^2+^ sites and participate in hydrogen bonding with MA^+^ cations, thereby passivating traps and suppressing non-radiative losses [86].

From a device perspective, these PL results are highly relevant. High steady-state PL under open-circuit-like conditions is characteristic of high-quality perovskite absorbers with low non-radiative recombination, which typically correlates with higher open-circuit voltage and improved power conversion efficiency. In operational devices, efficient charge extraction at short circuit leads to PL quenching; thus, the combination of strong PL in stand-alone films and strong quenching in complete devices is a hallmark of optimal absorber/transport-layer coupling [87]. The pronounced PL enhancement in the DMTH-containing films therefore foreshadows the superior photovoltaic performance and stability observed for these devices.

**Figure 11:**
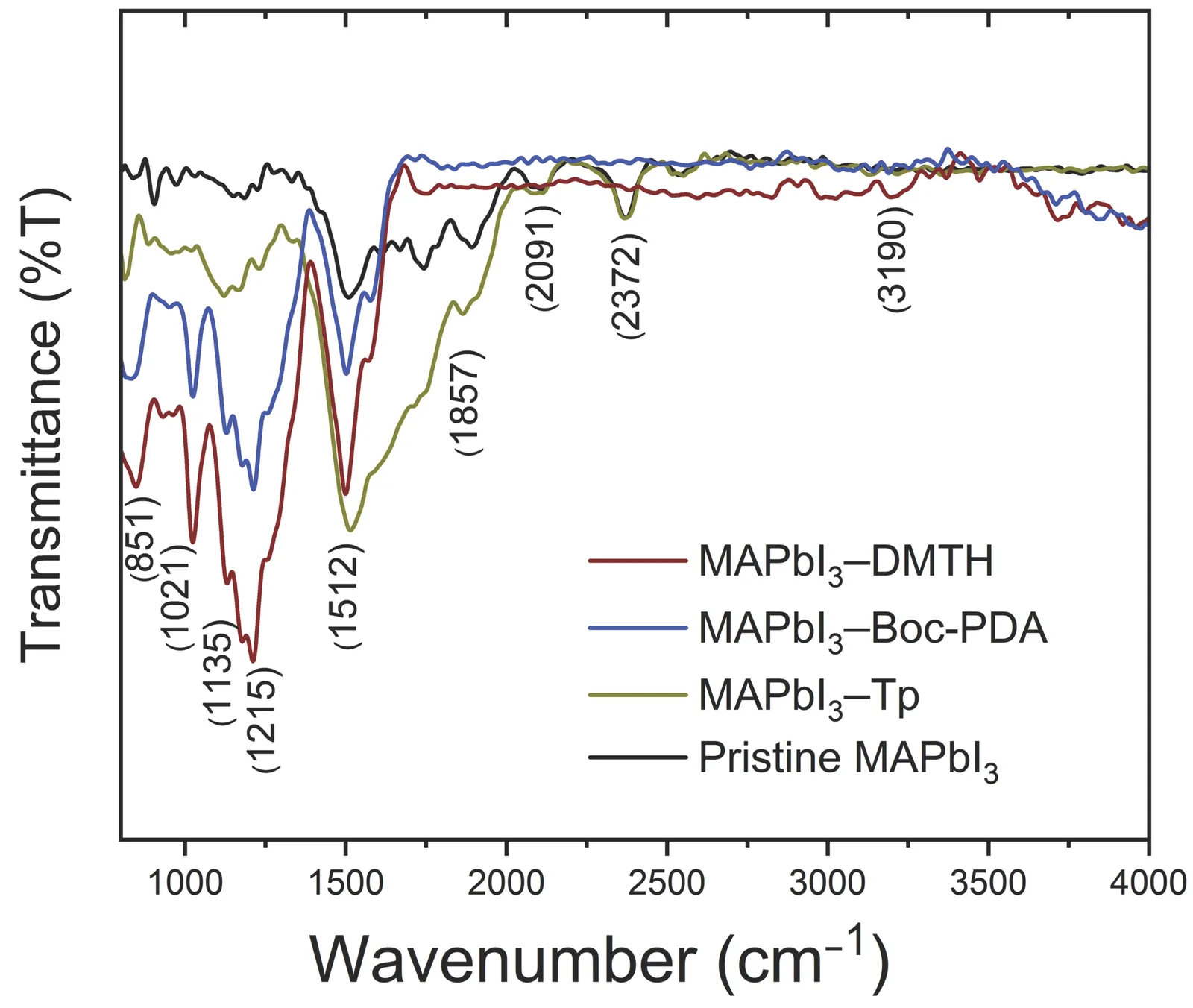
FTIR spectra of pristine MAPbI_3_ and COF-modified MAPbI_3_ films in the 975–4000 cm^−1^ region, highlighting the main vibrational modes of the organic sublattice and their evolution upon incorporation of DMTH, Boc-PDA, and Tp COFs.

Fourier-transform infrared spectroscopy was used to probe chemical interactions between the COFs (DMTH, Boc-PDA, and Tp-based frameworks) and the MAPbI_3_ lattice at the molecular level. Because the organic sublattice of MAPbI_3_ plays a central role in hydrogen bonding, ion migration, and structural stability [88], even subtle changes in the vibrational signatures of CH_3_NH_3_^+^ and COF functional groups can reveal how these additives reorganize the local bonding environment and help stabilize the perovskite. Figure 11 shows the FTIR spectra of pristine and COF-modified MAPbI_3_ films over the mid-IR range. The reference MAPbI_3_ film displays the expected vibrational fingerprint. The methylammonium (CH_3_–NH_3_^+^) rocking mode appears near 851 cm^−1^ [89], while prominent C–N stretching modes are observed at approximately 1021 and 1135 cm^−1^ [90]. A further band arising from the methylammonium cation is detected around 1215 cm^−1^, and a well-defined N–H bending mode of the ammonium group is located near 1512 cm^−1^ [46]. At higher wavenumbers, weak features are observed near 1857 and 2091 cm^−1^. Because the CH_3_NH_3_PbI_3_ lattice contains neither carbonyl nor nitrile functional groups, these features are not assigned to intrinsic C=O or C≡N stretching vibrations of the perovskite [91]. They appear at the same positions and with comparable intensity in all four spectra, including the pristine reference, and lie within the region in which the underlying glass substrate absorbs through Si–O overtone and combination modes [92]. They are therefore attributed to a residual contribution from the underlying glass substrate and are excluded from the structural interpretation of the films. The band near 2372 cm^−1^ is attributed to the asymmetric stretching vibration of atmospheric CO_2_ [93]. Its presence is consistent with the fact that no post-acquisition atmospheric correction was applied to the ATR-FTIR spectra.

A broad asymmetric N–H stretching envelope centered around 3190 cm^−1^ reflects the hydrogen-bonding environment of MA^+^ within the Pb–I framework [94]. Upon incorporation of COFs, these bands do not disappear or split into new impurity-related features, which indicates that the fundamental perovskite lattice is preserved. Instead, the spectra exhibit systematic shifts in peak positions and changes in relative intensity that can be directly linked to COF–perovskite interactions. All three COF-modified films show noticeable variations in the CH_3_–NH_3_^+^-related modes (rocking, bending, and C–H stretching) and in the C–O and C=O bands. For example, the N–H bending band near 1512 cm^−1^ slightly shifted and sharpened in the presence of COFs. These shifts are consistent with a change in the hydrogen-bonding strength between MA^+^ and surrounding iodide ions, as well as with additional hydrogen bonds and weak coordination involving COF functional groups such as hydrazides, Boc-protected amines, and aromatic carbonyls [95].

In the DMTH-modified film, for instance, the C–O and C=O bands show small but reproducible shifts and intensity changes, consistent with partial coordination of the hydrazide and methoxy groups to undercoordinated Pb^2+^ centers and with altered hydrogen-bonding networks involving MA^+^ and halides [96]. Similar, though less pronounced, trends are observed for Boc-PDA- and Tp-based films, indicating that all three COFs participate in interfacial interactions but with different strengths and geometries. The broad N–H stretching region around 3190 cm^−1^ is particularly informative. In pristine MAPbI_3_, the breadth of this band reflects a distribution of hydrogen-bond strengths between MA^+^ and iodide (N^+^–H···I) [97]. Any narrowing or shift in this band upon COF doping points to a more uniform and possibly stronger hydrogen-bonding environment, which is expected to suppress dynamic disorder of the organic cation and reduce the formation of defect-related local environments. The attenuation or modification of certain NH_3_^+^ stretching components in COF-containing samples can be interpreted as a consequence of additional N–H···O or N–H···N interactions introduced by the COFs, which partially redistribute the hydrogen-bonding network away from purely MA^+^–I^−^ contacts [95].

These molecular-level signatures are fully consistent with the broader structural and optoelectronic trends observed in this study. COF incorporation produces larger grains with lower microstrain and smoother, denser films, along with stronger band-edge emission, reduced non-radiative recombination, an unchanged bandgap and enhanced optical absorption. The FTIR evidence of modified CH_3_NH_3_^+^ and C–O/C=O vibrational environments provides the chemical basis for these observations: COFs interact with the perovskite at the grain boundaries and surfaces, coordinating to undercoordinated Pb^2+^ and participating in hydrogen bonding with MA^+^ and halides [98]. In doing so, they passivate traps, stabilize the organic–inorganic interface, and suppress vibrational and structural disorder, without disrupting the core PbI_6_ framework.

**Figure 12:**
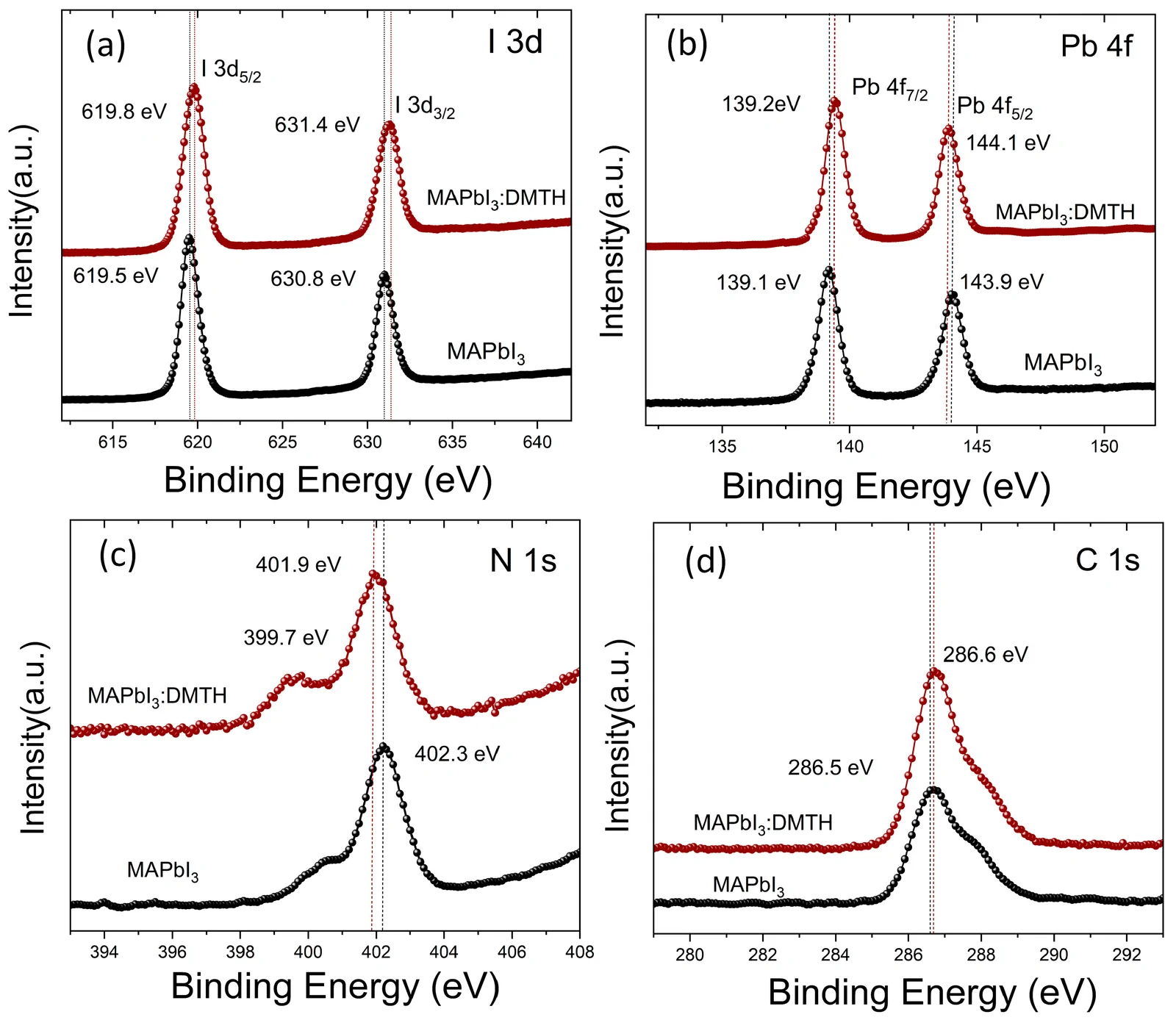
X-ray photoelectron spectra of pristine MAPbI_3_ and DMTH-COF-modified MAPbI_3_ films: (**a**) I 3d, (**b**) Pb 4f, (**c**) N 1s and (**d**) C 1s regions. The N 1s spectrum of the modified film shows an additional component at 399.7 eV, absent from the pristine reference, assigned to nitrogen of the incorporated framework.

X-ray photoelectron spectroscopy (XPS) was used to verify the presence of the DMTH-COF additive after thermal annealing and to evaluate its chemical interaction with the perovskite lattice. Figure 12 displays the high-resolution core-level spectra for both pristine and modified films. The pristine sample shows the I 3d doublet at 619.5 eV (I 3d_5/2_) and 630.8 eV (I 3d_3/2_). Incorporation of COF shifts these peaks to 619.8 eV and 631.4 eV, respectively, while the spin–orbit splitting remains approximately 11.6 eV [99]. The Pb 4f spectra exhibit a parallel shift: pristine peaks at 139.1 eV and 143.9 eV move to 139.2 eV and 144.1 eV in the modified film. The spin–orbit splitting stays at ~4.9 eV, and the absence of a signal near 136.8 eV confirms the strict preservation of the Pb^2+^ state [100]. The uniform shift in both Pb and I core levels, without changes to their splitting, indicates a surface Fermi level shift driven by the electron-donating COF rather than altered Pb–I covalency. The bulk halide sublattice therefore remains intact.

The N 1s spectra confirm the additive’s retention. The pristine film exhibits a single peak at 402.3 eV from the protonated methylammonium (MA^+^) cation [101]. After modification, the (MA^+^) peak is shifted to 401.9 eV and a clear second component appears at 399.7 eV. This new feature, 2.2 eV below the ammonium peak, is consistent with a less positively charged nitrogen environment and is therefore assigned to the hydrazide/hydrazone nitrogens in the DMTH framework [102]. Its presence confirms that the organic framework survives the 110 °C anneal. To decouple local chemical effects from uniform surface charging, the internal energy separation between the N 1s and I 3d_5_/_2_ core levels was evaluated. This gap increases by 0.7 eV, from 217.2 eV (pristine) to 217.9 eV (modified). This relative shift, combined with the displacement of the MA^+^ peak, shows that the additive chemically interacts with the perovskite rather than forming a segregated organic phase. The C 1s spectra show only a minor shift from 286.5 eV to 286.6 eV, as expected given the overlap between the dominant MA^+^ signal and the low-concentration COF (0.063 wt%). The XPS data establish that the DMTH-COF remains within the film, chemically couples to the perovskite at the surfaces or grain boundaries, and leaves the bulk Pb–I structural framework unperturbed. This surface-localized interaction supports the proposed passivation mechanism, explaining the suppressed non-radiative recombination without compromising the bulk lattice integrity.

**Figure 13:**
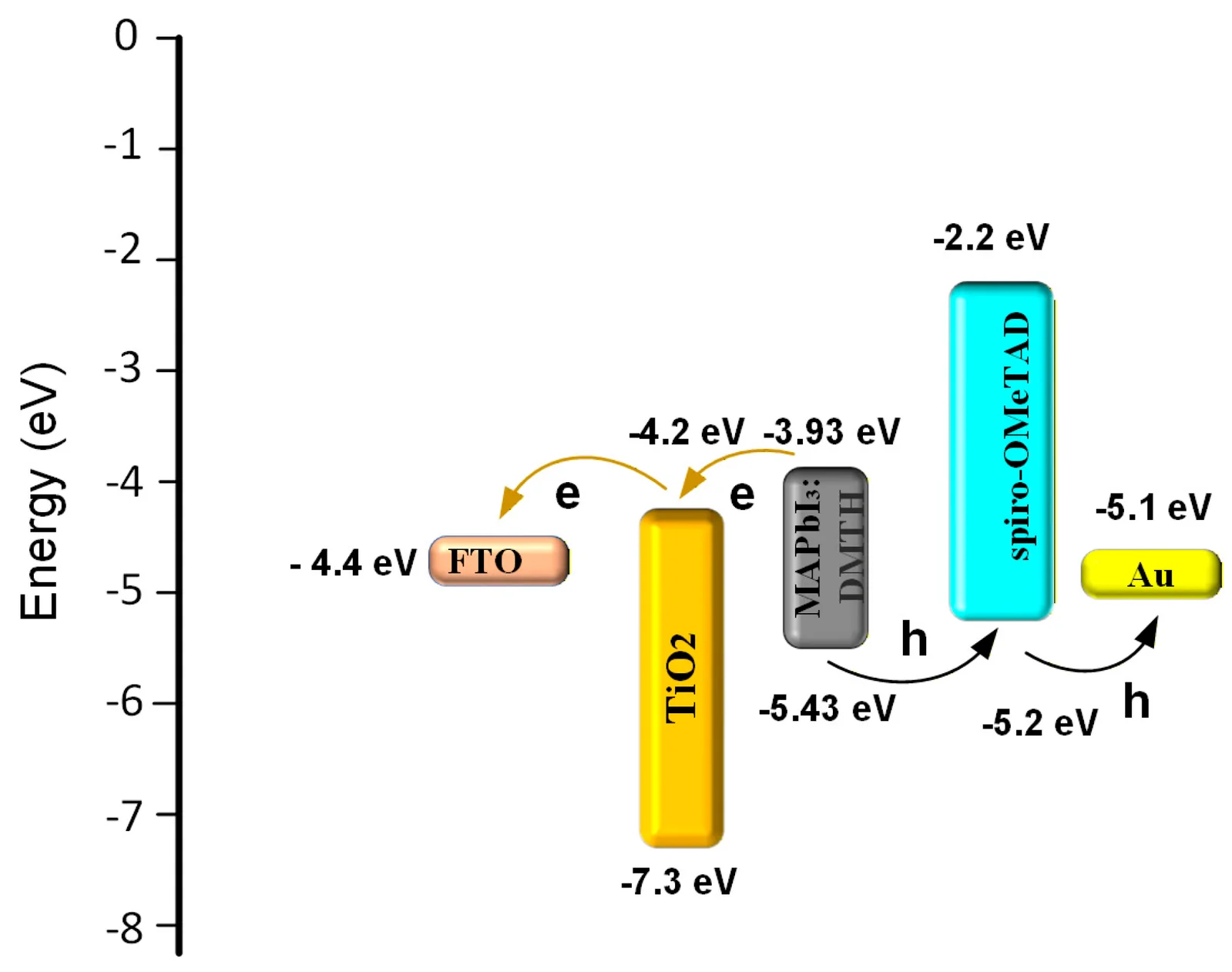
Schematic energy level diagram of the FTO/c-TiO_2_/m-TiO_2_/MAPbI_3_:DMTH-COF/Spiro-OMeTAD/Au device architecture, showing the electron- and hole-extraction pathways. The levels are taken from literature values and electrochemical estimates and are shown to illustrate the reference band alignment. No separate level is assigned to DMTH-COF, which is dispersed within the absorber rather than deposited as an interlayer.

The energy level diagram in Figure 13 illustrates the charge-extraction pathways in the perovskite solar cell, with a particular focus on the offsets along the hole-transport route. The energy levels shown in Figure 13 are based on literature values and electrochemical estimates rather than direct UPS/KPFM measurements, and are used to illustrate a plausible band-alignment scenario rather than to provide exact work-function values [103]. In the reference stack, the MAPbI_3_ conduction band minimum (CBM, ≈−3.93 eV) aligns reasonably well with the TiO_2_ CBM (≈−4.2 eV), which supports efficient electron extraction toward the FTO contact [104]. On the hole side, the MAPbI_3_ valence band maximum (VBM, ≈−5.43 eV) is offset from the Spiro-OMeTAD HOMO (≈−5.2 eV) and the Au work function (≈−5.1 eV) [105]. This offset of 0.23 eV between the perovskite VBM and the Spiro-OMeTAD HOMO is favorably oriented for hole extraction while remaining small enough to limit the associated energy loss.

DMTH-COF is incorporated within the MAPbI_3_ absorber rather than as a discrete layer and therefore does not contribute frontier levels to the charge-extraction pathway. The polar hydrazide and methoxy groups in DMTH-COF facilitate the formation of an oriented interfacial dipole at the perovskite surface, locally shifting the vacuum level and tuning the perovskite work function without significantly perturbing the bulk bandgap. This reduces the energy offset between the perovskite VBM and the Spiro-OMeTAD HOMO, which in turn lowers interfacial energy losses, enables a larger fraction of the photovoltage to be harvested, and supports a higher quasi-Fermi level splitting under illumination [106]. The alignment shown in Figure 13 is consistent with the experimental device trends. DMTH-containing cells exhibit higher Voc and fill factor than pristine devices, despite comparable optical bandgaps, indicating that fewer photogenerated carriers are lost to interfacial non-radiative recombination and that the built-in potential is more efficiently utilized. The microstructural advantages of DMTH-COF, comprising micrometer-scale grains, reduced microstrain and enhanced hydrophobicity, reduce the density of recombination-active sites within the absorber, so that a larger fraction of photogenerated holes reaches the Spiro-OMeTAD interface and is subsequently collected at the Au electrode. In parallel, electrons follow a separate, well-aligned route via TiO_2_ and FTO [107].

**Figure 14:**
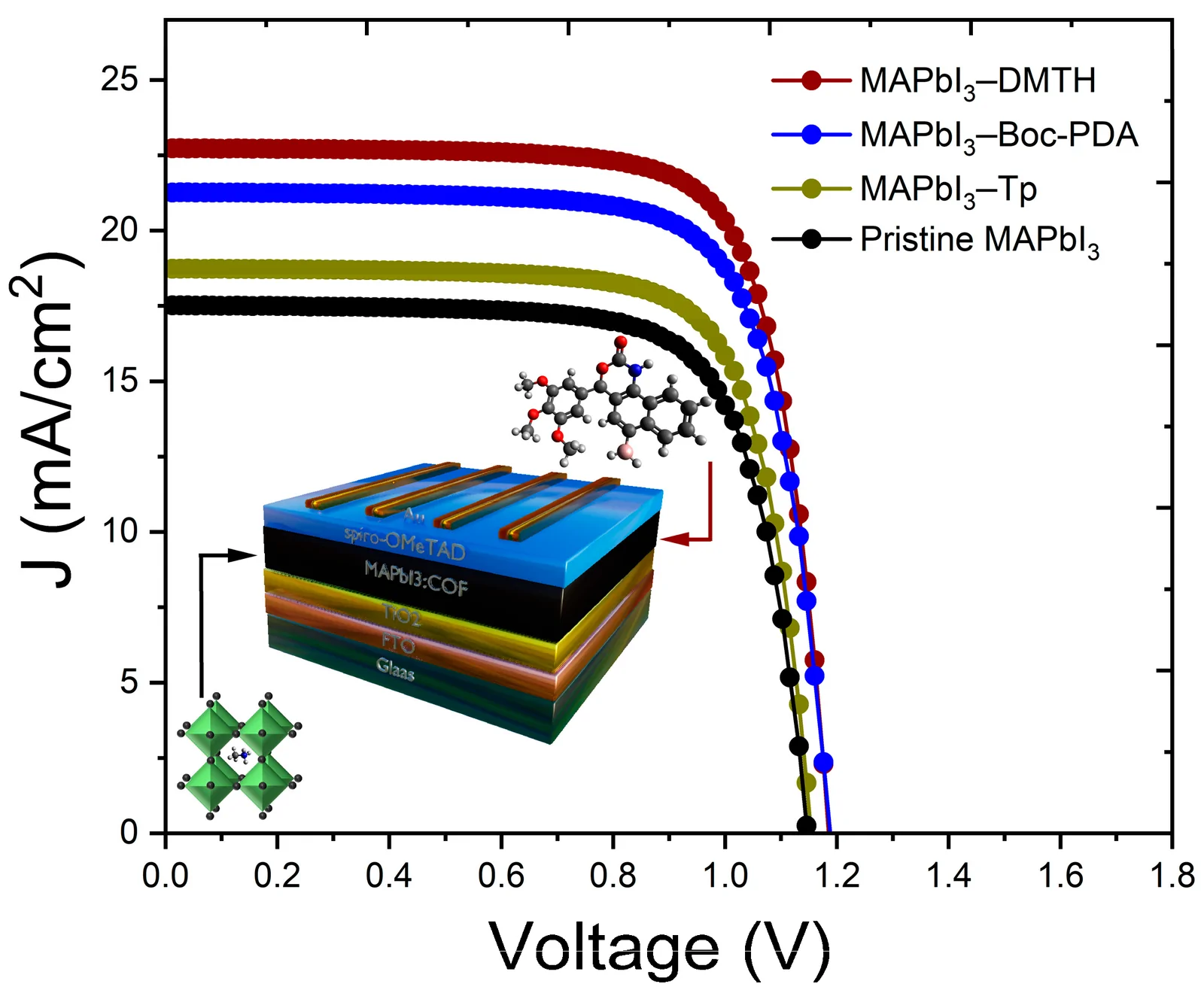
Current density–voltage (J–V) curves of MAPbI_3_ PSCs based on pristine and COF-modified absorbers (Tp-COF, Boc-PDA-COF and DMTH-COF), measured under AM 1.5G illumination (100 mW cm^−2^).

The photovoltaic performance of the devices provides an integrated assessment of how COF doping affects the structure, optoelectronic properties, and charge-transport characteristics of MAPbI_3_. PSCs were fabricated in a standard n–i–p configuration, glass/FTO/c–TiO_2_/m -TiO_2_/MAPbI_3_:COF/Spiro- OMeTAD/Au, using MAPbI_3_ absorbers prepared without and with COFs (Tp, Boc-PDA-phenylenediamine, and DMTH). All devices were measured under AM 1.5G illumination (100 mW cm^−2^). From Figure 14 and Table 3, the reference (undoped) MAPbI_3_ device exhibits a Voc of 1.142 V, a Jsc of 17.53 mA cm^−2^, a fill factor (FF) of 73.45%, and a power conversion efficiency (PCE) of 14.7%. These values are characteristic of solution-processed MAPbI_3_ cells limited by defect-mediated non-radiative recombination, incomplete light harvesting, and non-ideal series and shunt resistances [108]. Introduction of COF additives leads to a systematic improvement in all figures of merit. The Tp-modified device reaches 15.88% PCE, Boc-PDA-COF-modified devices reach 18.88%, and the DMTH-based device achieves the highest performance with Voc = 1.183 V, Jsc = 22.98 mA cm^−2^, FF = 76.46%, and PCE = 20.78%. This progression demonstrates that the extent of performance enhancement closely follows the effectiveness of each COF in controlling crystallization, passivating defects, and optimizing interfacial energetics.

**Table 3:** Photovoltaic parameters of MAPbI_3_ PSCs with and without COF doping, averaged over 10 devices under AM 1.5G illumination (100 mW cm^−2^). Rs and Rsh were extracted from the slopes of the J–V curves near Jsc and Voc, respectively.

Sample	V_oc_ (V)	Jsc (mA/cm^2^)	FF (%)	PCE (%)	Rs (Ω cm^2^)	R_sh_ (kΩ cm^2^)
Pristine	1.142 ± 0.002	17.53 ± 0.531	73.45 ± 1.246	14.7 ± 0.337	5.5	5.92
Tp	1.152 ± 0.003	18.75 ± 0.486	73.54 ± 1.434	15.88 ± 0.345	4.8	6.35
Boc-PDA	1.174 ± 0.005	21.33 ± 0.463	75.43 ± 1.484	18.88 ± 0.447	5.1	6.75
DMTH	1.183 ± 0.006	22.98 ± 0.461	76.46 ± 1.544	20.78 ± 0.37	4.0	7.74

The Voc enhancement from 1.142 V to 1.183 V reflects a reduction in non-radiative recombination losses in the bulk and at the interfaces. This is consistent with the strong PL intensity increase observed for COF-treated films, particularly for DMTH-COF, which indicates reduced trap-assisted recombination and higher quasi-Fermi level splitting. The Jsc increase, from 17.53 to 22.98 mA cm^−2^, arises from a combination of broader and more intense optical absorption and improved carrier collection [80]. UV–Vis spectra showed enhanced absorbance at an unchanged absorption onset for COF-doped films, while XRD and FE-SEM revealed enlarged grain sizes and fewer grain boundaries, both of which promote longer diffusion lengths and lower recombination probabilities. The largest Jsc in DMTH-based devices is therefore a direct manifestation of the most favorable combination of microstructure, defect passivation, and optical response. The fill factor also improves with COF doping, rising from 73.45% in the pristine device to 76.46% in the DMTH-COF case. This improvement is tightly linked to the evolution of series resistance (Rs) and shunt resistance (Rsh). The fill factor also improves with COF doping, rising from 73.45% in the pristine device to 76.46% in the DMTH-COF case. The series resistance extracted from the slope of the reverse-scan J–V curve at Voc falls from 5.5 Ω cm^2^ in the pristine device to 4.0 Ω cm^2^ in the DMTH-COF device, a reduction of 27%. The intermediate values obtained for the Tp-COF and Boc-PDA-COF devices, 4.8 and 5.1 Ω cm^2^, are close to the uncertainty of the extraction, so the comparison is made here between the pristine and DMTH-COF cases.

In parallel, Rsh increases from 5.92 to 7.74 kΩ cm^2^ with COF doping. Low Rsh in the pristine devices indicates the presence of leakage paths, such as micro-shorts, surface defects, and ion-migration-related shunts [108]. The higher Rsh in COF-modified devices shows that these parasitic channels are significantly suppressed. Morphologically, this corresponds to denser, more uniform films with reduced pinhole density, as seen by FE-SEM, and structurally to fewer extended defects and dislocations, as suggested by the narrowed XRD peaks and reduced microstrain. The concurrent decrease in Rs and increase in Rsh demonstrates that COF additives simultaneously enhance charge-transport pathways and block undesirable leakage routes, which together raise the FF and overall PCE.

The relative performance of the three COFs aligns with their structural and electronic characteristics. Tp-based COFs form ordered, β-ketoenamine networks with hexagonal pores that provide a basic scaffold for more uniform crystal growth and moderate passivation. Boc-PDA-derived COFs introduce aromatic and Boc-protected amine functionalities that more effectively interact with MA^+^ and Pb^2+^, leading to larger grains, lower trap density, and better surface passivation, and consequently a substantial jump in PCE to 18.88%. DMTH, containing methoxy-substituted hydrazide groups, goes further by combining strong coordination capability with electron-donating character and good solubility. This enables the formation of flexible, two-dimensional COF networks that intimately coat grain surfaces and interfaces, finely tuning crystallization.

**Figure 15:**
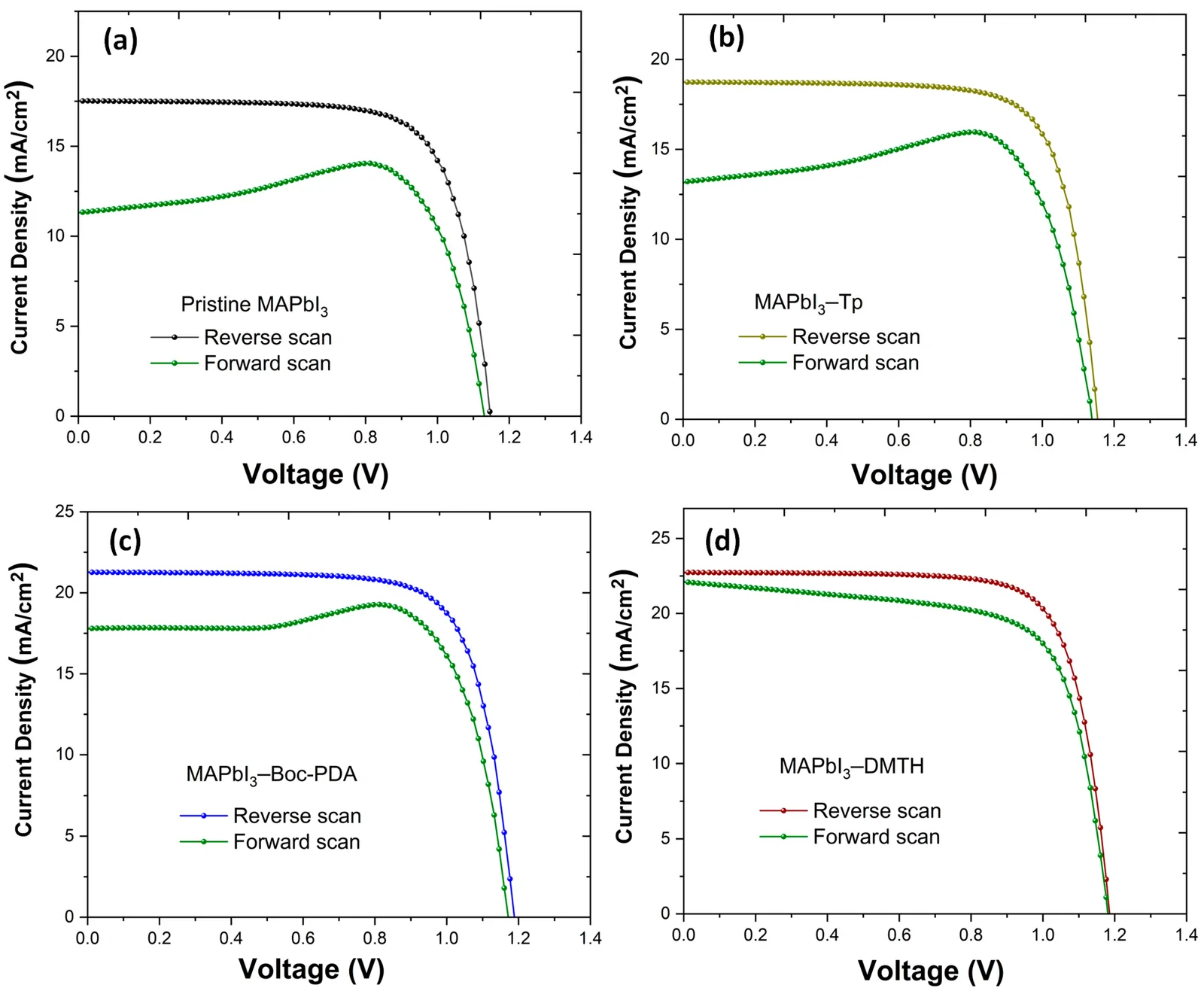
Forward and reverse current density–voltage, J–V, characteristics of MAPbI_3_ perovskite solar cells: (**a**) pristine MAPbI_3_, fabricated without an additive; (**b**) MAPbI_3_ with the Tp-COF additive; (**c**) MAPbI_3_ with the Boc-PDA-COF additive; (**d**) MAPbI_3_ with the DMTH-COF additive. In each panel the reverse and forward scans are shown. The corresponding hysteresis index values were calculated from the reverse- and forward-scan PCEs.

The photovoltaic parameters obtained from forward and reverse J–V sweeps, together with the calculated hysteresis indices, are summarized in Figure 15 and Table 4. The pristine MAPbI_3_ device shows pronounced scan dependence: its PCE falls from 14.84% in the reverse direction to 11.92% in the forward direction, giving a hysteresis index of 0.197. In MAPbI_3_ devices, such discrepancies generally arise from a combination of charge trapping, carrier accumulation at the contacts, and redistribution of mobile ionic species during the sweep [85]. Defects including undercoordinated Pb^2+^ sites and halide vacancies can promote all three processes by introducing trap states and distorting the local electric field.

**Table 4:** Reverse- and forward-scan photovoltaic parameters and the corresponding hysteresis indices of pristine and additive-modified MAPbI_3_ perovskite solar cells. The J–V characteristics were measured under simulated AM1.5G illumination at 100 mW cm^−2^, using identical scan conditions for all device configurations. The hysteresis index was calculated from the difference between the reverse- and forward-scan power conversion efficiencies according to HI = (PCE rev − PCE fwd)/PCE rev. The ΔJsc value represents the percentage decrease in short-circuit current density in the forward scan relative to the reverse scan. The reported values correspond to representative devices, while batch-averaged photovoltaic parameters obtained from 10 devices for each condition are summarized in Table 3.

Sample	PCE rev (%)	PCE fwd (%)	HI	ΔJsc (%)
Pristine-COF	14.84	11.92	0.197	35.3
Tp-COF	16.23	13.62	0.161	29.5
Boc-PDA-COF	18.86	16.85	0.107	16.3
DMTH-COF	20.38	18.13	0.110	2.7

All three additives reduce the scan dependence, though to different degrees. The hysteresis index drops to 0.161 with Tp, 0.107 with Boc-PDA, and 0.110 with DMTH. The improvement with Tp is modest, whereas Boc-PDA and DMTH nearly halve the hysteresis relative to the control. This suggests that both additives alter the defect and interfacial environment of the perovskite layer in ways that make charge extraction far less sensitive to sweep direction. The raw hysteresis index alone, however, does not tell the full story. Although Boc-PDA gives the marginally lower index, the DMTH device delivers the best absolute performance in both sweep directions, with PCEs of 20.38% and 18.13% under reverse and forward scans, respectively. Weighing hysteresis suppression against absolute efficiency, DMTH therefore offers the most favorable balance of the three additives.

The relative variation in Jsc between sweep directions falls monotonically from 35.3% in the pristine device to 29.5%, 16.3% and 2.7% for the Tp-, Boc-PDA- and DMTH-modified devices, indicating that photocurrent collection becomes substantially less dependent on scan direction once the additive is present. This is consistent with a lower trap density and reduced interfacial charge buildup [93]. A suppressed contribution from ion-migration-induced polarization is also plausible, although J–V sweeps alone cannot quantify ion motion; impedance spectroscopy or transient measurements would be needed to resolve that contribution directly. The observed trend is chemically reasonable. The nitrogen- and oxygen-bearing groups in these additives can coordinate undercoordinated Pb^2+^ sites or bind charged defects at grain boundaries and interfaces, lowering the density of electronically active traps and curbing non-radiative recombination. The particularly strong effect of DMTH plausibly reflects the arrangement of its hydrazide and methoxy groups, which provide multiple coordination sites within close proximity. We stress, however, that this remains a proposed mechanism: definitive confirmation would require direct spectroscopic or electrical evidence of the passivation interaction.

**Figure 16:**
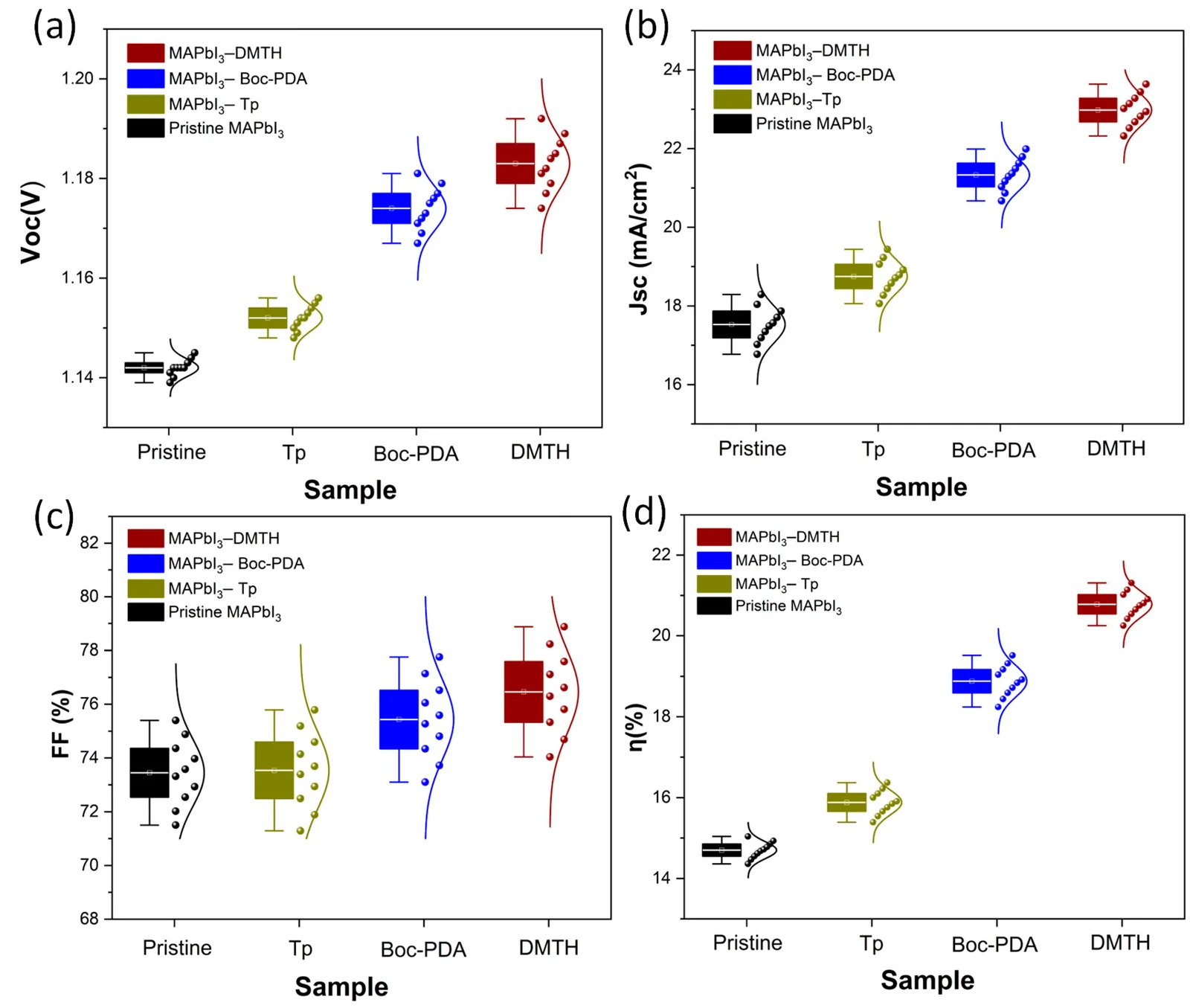
Statistical distributions of the photovoltaic parameters of pristine, Tp-, Boc-PDA-, and DMTH-modified MAPbI_3_ devices, extracted from (N = 10) devices per condition under AM 1.5G illumination (100 mW cm^−2^). Panels summarize the distributions of (**a**) open-circuit voltage (Voc), (**b**) short-circuit current density (Jsc), (**c**) fill factor (FF), (**d**) power conversion efficiency (PCE).

The reproducibility of the photovoltaic performance was evaluated using 10 independently fabricated devices for each condition (N = 10). As shown in Figure 16, the individual measurements were overlaid on the box plots to provide a direct representation of the device-to-device variation. The statistical values reported in Table 3 were calculated from the same datasets and are presented as the mean ± standard deviation. Figure 16 presents box plots of the solar cells that were examined in this study. The photovoltaic parameters of the pristine and COF-modified devices are presented as box plots. Figure 16a–d display the Voc in V, Jsc in mA cm^−2^, FF in%, and PCE in% of the solar cells, respectively. The mean Voc increased from 1.142 V for pristine samples to 1.183 V for the DMTH-COF device, a 3.59% increase. The Jsc enhancements are even more substantial, with an increase of 31.09% from 17.53 mA cm^−2^ for pristine to 22.98 mA cm^−2^ for the DMTH-COF device. The FF enhancements are also notable, with a pristine value of 73.45% and a DMTH-COF device value of 76.46%, resulting in a 4.10% increase in value. The increase in PCE is even more significant, with a rise from 14.7% for pristine to 20.78% for the DMTH-COF device, resulting in a 41.36% increase. The DMTH-modified devices achieved a mean PCE of 20.78 ± 0.37%, owing to the increases in Jsc, Voc, and FF. The ability of the COFs to passivate defects and improve charge-carrier mobility partially accounts for the increase in Jsc [109]. Furthermore, the best optimal Voc performance for devices is achieved through the reduction in recombination losses following the incorporation of these COFs, aligning with the PL and FTIR observations of decreased non-radiative pathways and improved stability under environmental stress.

**Figure 17:**
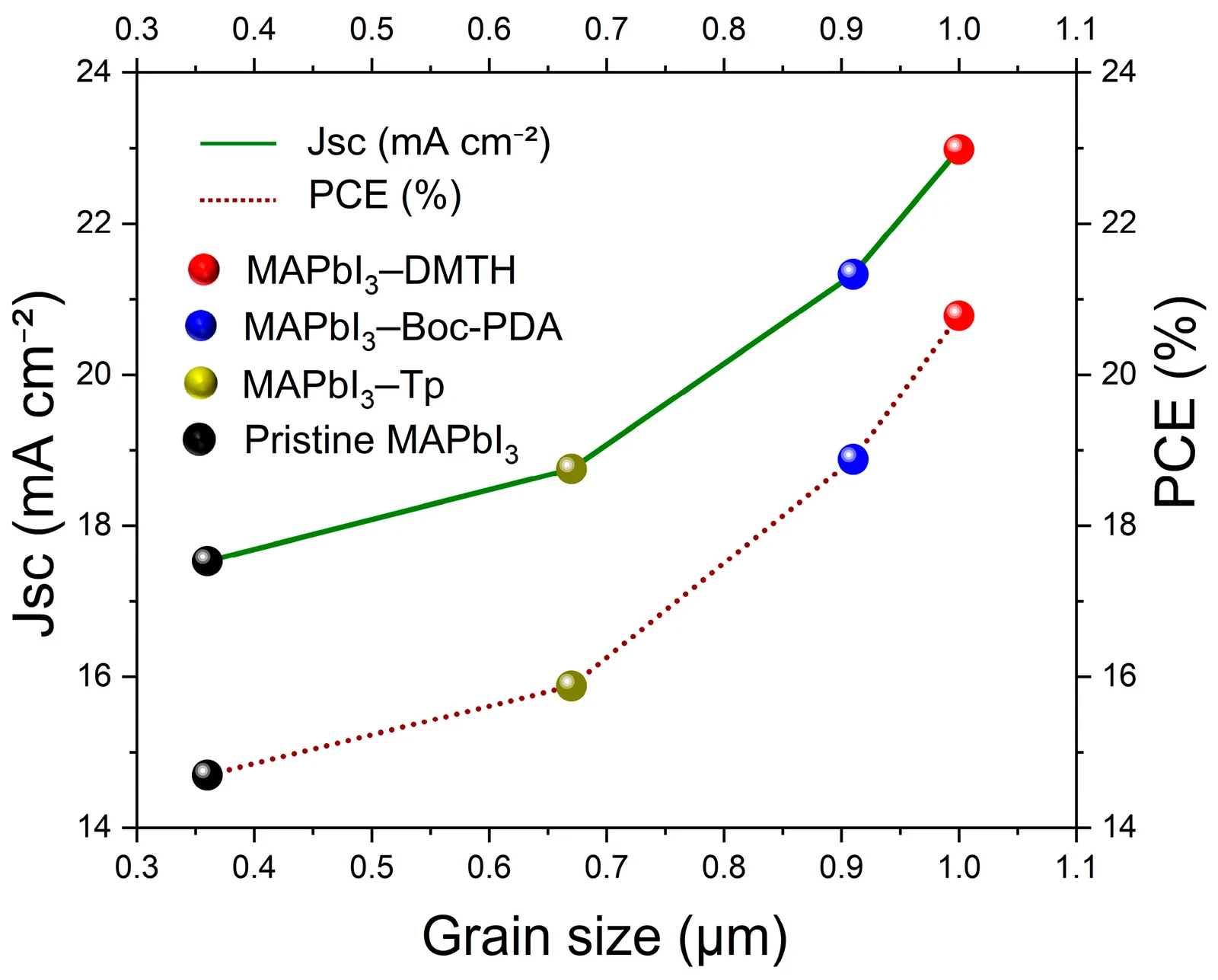
Correlation between mean grain size and device performance for pristine and COF-modified MAPbI_3_ perovskite solar cells. Short-circuit current density (Jsc, solid green line, left axis) and power conversion efficiency (PCE, dotted brown line, right axis) are plotted as a function of average grain size for pristine, Tp-, Boc-PDA-, and DMTH-doped MAPbI_3_ films. Both Jsc and PCE increase monotonically with grain size, demonstrating that COF-assisted grain enlargement and reduced grain-boundary density directly translate into enhanced charge collection and overall device efficiency.

To quantitatively link the microstructural evolution induced by COF doping with photovoltaic performance, we correlated the mean grain size extracted from FE-SEM analysis with the Jsc and PCE values obtained from J–V measurements. Figure 17 summarizes this relationship for pristine MAPbI_3_ and for films doped with Tp, Boc-PDA, and DMTH. The *x*-axis reports the average grain size for each composition (approximately 0.36 µm for pristine, 0.67 µm for Tp-COF, 0.91 µm for Boc-PDA-COF, and 1.00 µm for DMTH-COF), while the left and right y-axes correspond to Jsc and PCE, respectively. Both Jsc (solid green line) and PCE (dotted brown line) increase monotonically as the grain size grows. The pristine film, with the smallest grains, exhibits the lowest Jsc (17.53 mA cm^−2^) and PCE (14.7%). Introducing Tp-COF leads to moderate grain enlargement and a corresponding increase in Jsc and PCE. Boc-PDA-COF further pushes the grain size close to 0.9 µm and yields a more pronounced rise in both parameters. The DMTH-doped film, which achieves grains near 1.0 µm, delivers the highest Jsc (22.98 mA cm^−2^) and PCE (20.78%). This trend indicates that enlarging the perovskite grains and reducing the density of grain boundaries are key to improving charge collection and suppressing recombination losses [110]. The correlation in Figure 17 directly supports the microstructure–property–performance linkage established throughout the study. Larger grains formed under COF-assisted crystallization reduce the number of grain boundaries per unit volume, thereby lowering the density of boundary-related traps and non-radiative recombination centers [111]. This structural refinement enables photogenerated carriers to traverse longer distances without recombination, which manifests as increased Jsc and higher internal quantum efficiency. At the device level, this improved collection efficiency, combined with reduced series resistance and higher shunt resistance in COF-doped devices, drives the observed PCE enhancement.

**Figure 18:**
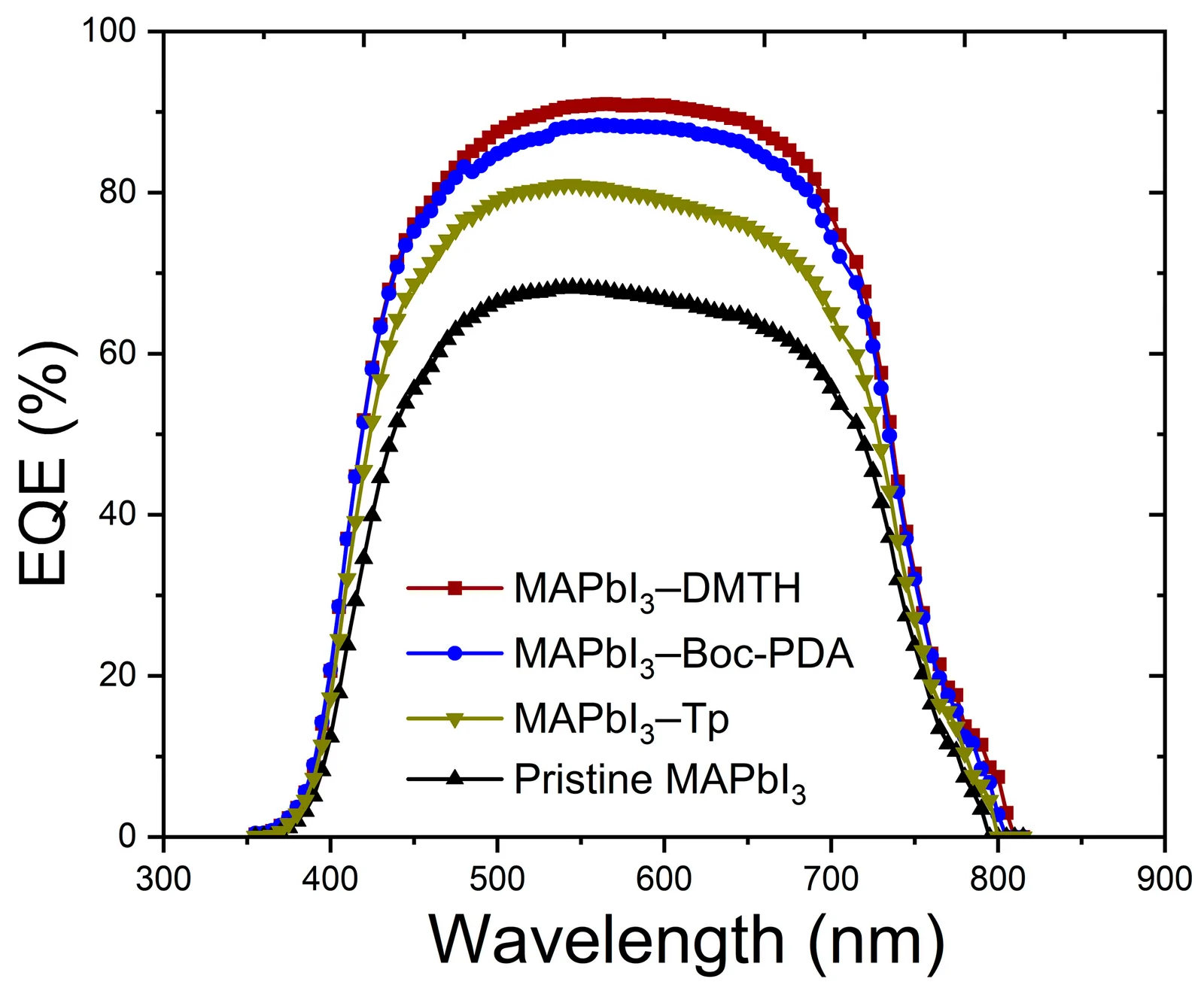
External quantum efficiency (EQE) spectra of MAPbI_3_ PSCs fabricated with pristine and COF-doped absorbers (Tp-COF, Boc-PDA-COF, and DMTH-COF), measured in the 300–900 nm range under monochromatic illumination.

Figure 18  shows the external quantum efficiency (EQE) spectra of PSCs based on pristine MAPbI_3_ and COF-modified absorbers (Tp, Boc-PDA, and DMTH), recorded under monochromatic illumination in the 300–900 nm range. The pristine device exhibits a maximum EQE of approximately 68% in the visible region (about 400–800 nm), with a pronounced roll-off toward longer wavelengths and an integrated Jsc of around 17.5 mA cm^−2^, in agreement with the J–V data. The COF-doped devices display progressively higher and broader EQE responses, with the Tp- and Boc-PDA-COF-modified cells already showing enhanced conversion efficiency across most of the spectrum and reduced loss in the red/near-IR region. The DMTH-based device reaches a peak EQE close to 92% and maintains a strong response up to about 800 nm, corresponding to an integrated Jsc of approximately 23.0 mA cm^−2^.

The evolution of the EQE spectra mirrors the structural and optoelectronic improvements induced by COF incorporation. The higher EQE in the blue–green region reflects more effective photogeneration and collection in the bulk perovskite, consistent with the enlarged grains, reduced microstrain, and improved crystallinity seen in XRD and FE-SEM. The sustained EQE at longer wavelengths for the COF-treated devices, particularly DMTH-COF, indicates that carriers generated deeper in the absorber experience fewer trap-assisted losses and can be extracted more efficiently, in line with the enhanced PL intensity and improved carrier collection inferred for these films. The good agreement between the EQE-integrated Jsc values and those obtained from J–V curves indicates that the COF additives do not introduce significant parasitic absorption or optical losses in the working spectral range. This, together with the enhanced PL and device performance, is consistent with an improvement in internal quantum efficiency through suppressed recombination and optimized charge transport throughout the device stack.

**Figure 19:**
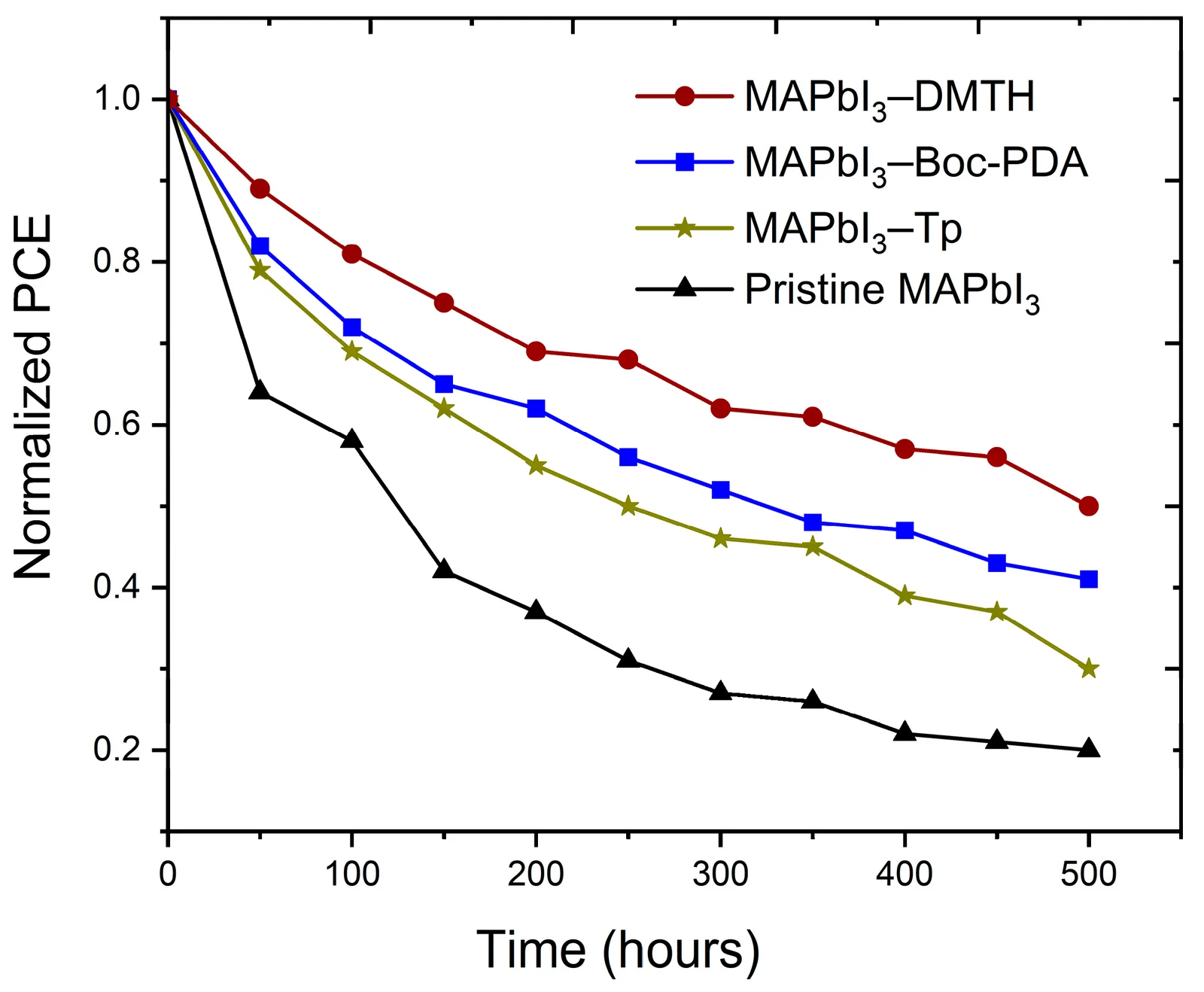
Stability of pristine and COF-modified MAPbI_3_ PSCs under continuous AM 1.5G illumination (100 mW cm^−2^) at 25 °C and 50% relative humidity for 500 h. The plots show the normalized evolution of PCE for devices based on pristine MAPbI_3_ and MAPbI_3_ doped with Tp, Boc-PDA, and DMTH.

Figure 19 presents a quantitative comparison of the operational stability of pristine and COF-modified MAPbI_3_ PSCs over 500 h of continuous AM 1.5G illumination (100 mW cm^−2^) at 25 °C and 50% relative humidity. This accelerated stress test directly probes the susceptibility of the different absorber formulations to photo- and moisture-induced degradation and links the microstructural and interfacial improvements induced by COFs to long-term device performance. The pristine MAPbI_3_ devices start from Voc = 1.142 V, Jsc = 17.53 mA cm^−2^, FF = 73.45%, and PCE = 14.7% but deteriorate rapidly under continuous operation. After 500 h, they retain only about 20% of their initial PCE (final PCE ≈ 2.94%). This steep decline is characteristic of unprotected MAPbI_3_ and can be attributed to the combined effects of moisture ingress and light-induced ion migration [112]. Water molecules diffuse along grain boundaries and interfaces, facilitating hydrolysis of the methylammonium cation, PbI_2_ segregation, and progressive formation of non-perovskite phases [113]. At the same time, the high density of unpassivated defects at grain boundaries promotes ion migration and interfacial reactions, thereby intensifying non-radiative recombination. These processes manifest macroscopically as a rapid loss of Voc (increased non-radiative losses) and FF (increased series and leakage components), with only modest changes in Jsc as the absorber progressively decomposes.

Incorporation of COFs significantly alters this degradation pathway. DMTH-doped devices, which initially deliver Voc = 1.183 V, Jsc = 22.98 mA cm^−2^, FF = 76.46%, and PCE = 20.78%, show a noticeably slower performance decay. After 500 h, they retain approximately 50% of their initial PCE, corresponding to a final PCE of approximately 10.39%. The more gradual loss of Voc and FF indicates that DMTH-COF reduces the formation and activation of non-radiative recombination centers during operation. This behavior is consistent with the structural and spectroscopic evidence that DMTH-derived COFs passivate undercoordinated Pb^2+^ and halide sites through coordination and hydrogen bonding, enlarge grains, and reduce microstrain. The resulting reduction in defect density and ion-migration pathways at grain boundaries delays the onset of moisture-driven decomposition and preserves more of the built-in potential under stress. Devices doped with Boc-PDA-COF also exhibit markedly improved stability relative to pristine cells. Their initial performance (Voc = 1.174 V, Jsc = 21.33 mA cm^−2^, FF = 75.43%, PCE = 18.88%) translates into a PCE retention of about 41% after 500 h (final PCE ≈ 7.74%). The Boc-PDA protected amine groups in this COF provide a combination of aromatic conjugation and steric protection at the perovskite surface and grain boundaries, which enhances interfacial adhesion, suppresses the evaporation of volatile organic species, and passivates traps. As a result, the evolution of leakage paths and shunt channels is slowed, and the FF remains relatively high even after extended operation. Among the three COF-modified devices. Starting from Voc = 1.152 V, Jsc = 18.75 mA cm^−2^, FF = 73.54%, and PCE = 15.88%, they maintain approximately 30% of their original PCE after 500 h (final PCE ≈ 4.76%). The relatively superior stability of Tp-containing cells reflects the robust, β-ketoenamine-type network formed by Tp-based COFs, which provides a mechanically and chemically stable framework around perovskite grains. This framework likely enhances lattice rigidity, reduces stress-induced phase transitions, and offers an effective barrier against moisture penetration along grain boundaries. The high PCE retention factor (0.50 at 500 h, compared to 0.20 for pristine) directly evidences this stabilizing effect.

The stability trends across pristine, Tp-, Boc-PDA-, and DMTH-doped devices mirror the way each COF modifies crystallization, defect chemistry, and surface wettability. All three COFs enlarge grains, reduce microstrain, and increase water contact angles relative to pristine MAPbI_3_, thereby limiting the density of reactive sites and slowing water ingress. At the same time, their distinct functional groups (hydrazide and methoxy in DMTH, Boc-protected amines in Boc-PDA, and keto-enolic motifs in Tp) tailor the balance between passivation strength, lattice rigidity, and interfacial protection. After 500 h, the normalized PCE retention values were approximately 0.20 for pristine MAPbI_3_, 0.50 for DMTH-COF, 0.41 for Boc-PDA-COF, and 0.30 for Tp-COF. Thus, DMTH-COF produced the highest initial efficiency, whereas Tp-COF provided the highest relative stability under the applied aging conditions. The improved stability of the COF-modified devices is consistent with reduced defect density, modified film microstructure, and increased surface hydrophobicity, although the individual contributions of defect passivation, ion migration, and moisture transport require further direct investigation.

Table 5  compares the photovoltaic performance and stability of the present devices with previously reported COF-modified and additive-engineered perovskite solar cells. The DMTH-containing device achieved a PCE of 20.78%, close to the 20.04% reported for a triazine-based COF additive in MAPbI_3_ and the 21.35% obtained using MAOCN in the same absorber system. Higher efficiencies have been reported for an amino-functionalized COF in a mixed-cation perovskite (22.13%) and for dual-interface COF modification (24.26%). These values, however, should not be compared solely on the basis of efficiency because the studies differ in absorber composition, device architecture, active area, and measurement conditions. In particular, mixed-cation perovskites generally offer greater compositional flexibility and improved thermal stability compared with conventional MAPbI_3_.

Among the MAPbI_3_ devices listed in Table 5, the DMTH-modified cell exhibits the highest open-circuit voltage, reaching 1.183 V compared with 1.04 and 1.126 V in the two most closely related additive studies. This result agrees with the stronger photoluminescence observed for the DMTH-containing film, which suggests that non-radiative recombination was reduced. By contrast, its fill factor of 76.5% remains below the previously reported values of 79.4% and 81.2%. The larger active area used in this work (0.50 cm^2^) may have contributed to this difference through increased series resistance and current-collection losses, although variations in device architecture and fabrication conditions may also be involved. The stability data must likewise be interpreted in relation to the aging conditions. Most of the reference devices were evaluated during dark storage under ambient or controlled-humidity conditions. In the present study, the devices were exposed to continuous AM1.5G illumination at 50% relative humidity. Continuous illumination can activate degradation processes that are absent or less pronounced during dark storage, including photoinduced ion redistribution, interfacial deterioration, and accelerated decomposition in the presence of moisture. The reported retention values are therefore not directly equivalent. Although the DMTH device does not provide the highest efficiency in the broader comparison, its performance under a more demanding illumination-and-humidity protocol demonstrates the practical value of introducing DMTH directly into the MAPbI_3_ precursor solution.

## 4. Conclusions

This work establishes covalent organic framework (COFs) as a powerful and versatile class of bulk dopants for engineering MAPbI_3_ perovskite thin films and devices. By integrating three structurally distinct frameworks, DMTH-COF, Boc-PDA-COF and Tp-COF, each prepared from commercially available building blocks, directly into the MAPbI_3_ precursor solution, we demonstrate that COFs can act simultaneously as crystallization regulators and defect passivators. Unlike most previous reports where COFs are confined to surface passivation layers or used as inert scaffolds, our approach uses COFs as bulk additives to tune film formation and electronic structure in a single solution-processing step, without sacrificing phase purity or process simplicity. Structurally, XRD and FE-SEM show that COF incorporation preserves the tetragonal MAPbI_3_ phase while driving a profound reorganization of the microstructure. Grain sizes increase from a few hundred nanometers in pristine films to approximately one micrometer in DMTH-modified layers, with concomitant narrowing of diffraction peaks and reduced microstrain. Raman and FTIR analyses reveal that this morphological improvement is underpinned by specific chemical interactions: COF functional groups coordinate undercoordinated Pb^2+^ sites and participate in hydrogen bonding with MA^+^ and halides, which relieves local lattice strain, stabilizes the organic sublattice, and suppresses vibrational disorder. These molecular-scale interactions directly reduce the density of electronic traps at grain interiors and boundaries. Optically and electronically, COF doping enhances light harvesting and carrier management without compromising the intrinsic perovskite band structure. Tauc analysis performed with a common background subtraction and a common extrapolation range gives optical bandgaps equal to within 7 meV across all four films at 1.56 eV, consistent with the invariant photoluminescence emission energy and the common EQE absorption onset. Steady-state PL and EQE measurements confirm that COF-treated films exhibit higher radiative efficiency and improved photon-to-current conversion, particularly at longer wavelengths where recombination losses are typically severe. Water contact angle measurements further show that COF doping, especially with DMTH, renders the film surface significantly more hydrophobic, limiting moisture ingress and reinforcing environmental stability at the most vulnerable interfaces. To our knowledge, this is the first systematic comparison of three chemically distinct COFs on MAPbI_3_, directly correlating COF structure with grain growth, defect passivation, surface wettability, and spectral response within a single, consistent device platform. X-ray photoelectron spectroscopy establishes that the framework survives annealing and remains chemically coupled to the perovskite. At the device level, these coupled structural and optoelectronic improvements translate into a clear and statistically robust progression in all key photovoltaic metrics. Relative to pristine MAPbI_3_ cells, which reach about 14.7% PCE, COF-doped devices exhibit systematically higher Voc, Jsc, FF, and PCE, with the DMTH-based cells achieving Voc = 1.183 ± 0.006 V, Jsc = 22.98 ± 0.46 mA cm^−2^, FF = 76.46 ± 1.544%, and PCE = 20.78 ± 0.37%. These gains are accompanied by a marked reduction in series resistance and an increase in shunt resistance, reflecting improved grain connectivity, lower trap density, fewer leakage paths, and a more favorable interfacial energy-level alignment inferred from the measured device performance and literature energy levels. Box-plot statistics show that DMTH not only lifts the average performance but also reduces device-to-device variations, which is essential for scalable fabrication. In contrast to more conventional dopants such as simple salts or polymers, the COFs used here deliver concurrent improvements in morphology, energetics, and moisture resistance, highlighting a genuinely multifunctional and previously under-explored design space for perovskite optimization.

## Figures and Tables

**Table 1:** Lattice parameters, FWHM, crystallite size (D), and microstrain (ε) of MAPbI_3_ thin films, pristine and doped with different COF systems.

Sample	a = b (Å)	c (Å)	FWHM (°)	D (nm)	ϵ (×10^−3^)
Pristine MAPbI_3_	8.8945	12.7577	0.23	35.6	3.56
Tp-COF	8.8942	12.7573	0.18	45.49	2.76
Boc-PDA-COF	8.8953	12.7585	0.17	48.17	2.53
DMTH-COF	8.8957	12.7583	0.15	54.59	2.26

**Table 2:** Lorentzian-fitted positions of the principal MAPbI_3_ lattice modes and their shifts relative to the pristine film.

Sample	54 m^−1^ Mode	Shift	112 cm^−1^ Mode	Shift
Pristine	58.22 ± 0.50	—	121.88 ± 0.20	—
Tp-COF	55.97 ± 0.14	−2.25	119.93 ± 0.22	−1.95
Boc-PDA-COF	55.44 ± 0.18	−2.78	118.17 ± 0.40	−3.71
DMTH-COF	54.71 ± 0.14	−3.51	115.88 ± 0.09	−6.00

**Table 5:** Comparison of the present work with reported COF-modified and additive-engineered perovskite solar cells.

Additive and Role	Perovskite	Architecture	Voc (V)	Jsc (mA cm^−2^)	FF (%)	PCE (%)	Stability Test	Retention	Ref.
NH2-COF, additive	Cs/FA/MA mixed	n-i-p	1.148	24.94	77.3	22.13	1000 h, 30 °C, 45–55% RH, dark	81%	Gao 2024 [15]
TPBTz-COF, bulk additive	MAPbI3	inverted p-i-n	1.04	24.26	79.4	20.04	400 h, 30 °C, 60% RH, dark	80%	Wu 2024 [114]
HS-COF, dual-interface layer	mixed cation	n-i-p	—	—	—	24.26	2000 h, 25 °C, 35–45% RH, dark	88%	Guo 2023 [30]
MAOCN, bulk additive	MAPbI3	n-i-p	1.126	23.37	81.2	21.35	30 days, ambient, dark	~95%	Tao 2021 [108]
DMTH, bulk additive	MAPbI3	mesoscopic n-i-p	1.183	22.98	76.5	20.78	500 h, 25 °C, 50% RH, continuous 1-sun illumination	50%	This work

## Data Availability

The data presented in this study are available upon request from the corresponding author. The data are not publicly available due to their inclusion in an ongoing research programme on additive engineering in MAPbI_3_ perovskite solar cells, with further findings being prepared for publication.
